# Disruption of Sorting Nexin 5 Causes Respiratory Failure Associated with Undifferentiated Alveolar Epithelial Type I Cells in Mice

**DOI:** 10.1371/journal.pone.0058511

**Published:** 2013-03-19

**Authors:** Sun-Kyoung Im, HyoBin Jeong, Hyun-Woo Jeong, Kyong-Tai Kim, Daehee Hwang, Machiko Ikegami, Young-Yun Kong

**Affiliations:** 1 School of Biological Science, College of Natural Sciences, Seoul National University, Seoul, South Korea; 2 Department of Life Science, Division of Molecular and Life Science, POSTECH, Pohang, South Korea; 3 School of Interdisciplinary Biosciences and Bioengineering, POSTECH, Pohang, South Korea; 4 Division of Pulmonary Biology, Cincinnati Children’s Hospital Medical Center and University of Cincinnati College of Medicine, Cincinnati, Ohio, United States of America; University of Illinois College of Medicine, United States of America

## Abstract

Sorting nexin 5 (Snx5) has been posited to regulate the degradation of epidermal growth factor receptor and the retrograde trafficking of cation-independent mannose 6-phosphate receptor/insulin-like growth factor II receptor. Snx5 has also been suggested to interact with Mind bomb-1, an E3 ubiquitin ligase that regulates the activation of Notch signaling. However, the *in vivo* functions of Snx5 are largely unknown. Here, we report that disruption of the *Snx5* gene in mice (*Snx5^-/-^* mice) resulted in partial perinatal lethality; 40% of *Snx5^-/-^* mice died shortly after birth due to cyanosis, reduced air space in the lungs, and respiratory failure. Histological analysis revealed that *Snx5^-/-^* mice exhibited thickened alveolar walls associated with undifferentiated alveolar epithelial type I cells. In contrast, alveolar epithelial type II cells were intact, exhibiting normal surfactant synthesis and secretion. Although the expression levels of surfactant proteins and saturated phosphatidylcholine in the lungs of *Snx5^-/-^* mice were comparable to those of *Snx5^+/+^* mice, the expression levels of T1α, Aqp5, and Rage, markers for distal alveolar epithelial type I cells, were significantly decreased in *Snx5* ^-/-^ mice. These results demonstrate that *Snx5* is necessary for the differentiation of alveolar epithelial type I cells, which may underlie the adaptation to air breathing at birth.

## Introduction

During lung development, the proximal-distal axis is formed by the elongation, expansion, and bifurcation of lung buds during branching morphogenesis, and the functional units for gas exchange are generated during alveologenesis. During late gestation, alveolar development occurs and type I and type II alveolar epithelial cells undergo differentiation and maturation to form functional alveoli [1], [2], [3], [4], [5], [6]. Alveolar epithelial type I cells are in close contact with endothelial cells in alveolar capillaries and form an efficient gas exchange area [7]. Alveolar epithelial type II cells undergo marked biochemical and ultrastructural changes, including depletion of glycogen content, enhanced synthesis of surfactant proteins and lipids, increased numbers of lamellar bodies, and surfactant secretion. In neonates, deficiency of surfactant protein production or secretion due to pulmonary immaturity can cause clinical disorders, such as respiratory distress syndrome or bronchopulmonary dysplasia. Respiratory distress syndrome is a major cause of high morbidity and mortality in premature infants [8], [9], [10].

Deletion or mutation of genes encoding surfactant proteins (SP)-B, SP-C, and ATP-binding cassette (ABC) A3 (ABCA3) in mice causes respiratory failure or severe lung disease soon after birth [11], [12], [13], [14]. In addition, recently, mutant mice with knock-in of the thyroid hormone receptor repressor silencing mediator of retinoid and thyroid hormone receptors (SMRT) have provided a novel model for alveolar epithelial type I cell-associated respiratory distress syndrome [15]. Although functional maturation of alveolar epithelial type I and II cells is critical for the adaptation of air breathing at birth, the molecular mechanisms that control the functional maturation of alveolar epithelial cells, particularly type I cells, are poorly understood [16], [17].

Sorting nexins (SNXs) are a large family of proteins that contain the phosphoinositide-binding Phox homology domain. Previous studies have reported that SNX5 plays a role in endosomal trafficking of epidermal growth factor receptor (EGFR) and cation-independent mannose 6-phosphate receptor (CI-MPR)/insulin-like growth factor II receptor (IGF2R) [18], [19], [20], [21]. Overexpression of SNX5 inhibits the degradation of EGFR, whereas the overexpression of SNX1 or SNX6 enhances EGFR degradation, suggesting that EGFR degradation may be finely regulated by SNX1, SNX5, and SNX6 [18], [22], [23]. Knockdown experiments of SNX5 and SNX6 using RNA interference (RNAi) show dispersed CI-MPR trafficking in HeLa cells into early endosomes [20], suggesting that CI-MPR is also regulated by SNX5 in endosome-to-trans-Golgi network (TGN) retrieval [24]. In addition, SNX5 interacts with Mind bomb-1 (Mib1), a key regulator of Notch ligands in mammalian development [25], [26], [27], [28], [29], [30], [31]. In zebrafish, depletion of *snx5* by morpholino leads to severe defects in vascular formation and hematopoietic cell generation [21], suggesting that *Snx5* may be involved in Notch signaling through regulation of *Mib1*. Despite a decade of studies examining the role of SNX5, the precise physiological function of SNX5 in mammals is still not known.

To study the *in vivo* functions of SNX5, we generated mice lacking the *Snx5* gene. These *Snx5^-/-^* mice showed partial perinatal mortality, with approximately 40% of the animals dying within a day of birth. Thirty percent of *Snx5^-/-^* mice survived until adulthood, but displayed severe growth inhibition. Downstream signaling of EGFR, EGFR degradation and endosome-to-TGN retrieval of CI-MPR were not affected in *Snx5^-/-^* murine embryonic fibroblasts (MEFs). In addition, EGFR signaling was not altered in isolated primary lung epithelial cells (PLEs). Neonatal *Snx5^-/-^* mice showed significant breathing defects associated with cyanosis and reduced pulmonary air space in the lungs at birth. Histological analysis revealed that *Snx5^-/-^* mice had reduced alveolar epithelial type I cells, while alveolar epithelial type II cells were intact. Consistently, *Snx5^-/-^* mice had decreased levels of the alveolar epithelial type I cell markers T1α, Aqp5, and Rage, whereas the expression levels of surfactant proteins and saturated phosphatidylcholine were comparable to wild-type mice. The results of our study indicate that *Snx5* is necessary for the differentiation of alveolar epithelial type I cells during perinatal murine lung development.

## Materials and Methods

### Ethics statement

Mice used in this study were housed under specific pathogen-free conditions. All animal protocols were approved by the Seoul National University Institutional Animal Care and Use committee (Approval number: SNU080623-4).

### Mouse experiments

The XA155 gene-trapped ES cell line, which contained an insertion of β-geo in intron 7 of *Snx5*, was used to generate chimeric mice (BayGenomics, San Francisco, CA). Following a standard protocol (http://www.genetrap.org/info/protocols/baygenomics/blastocyst.html), XA155 ES cells were microinjected into C57BL/6 mouse blastocysts. The resultant chimeric mice were mated with C57BL/6 mice to obtain heterozygous *Snx5^+/-^* mice. The heterozygous mutant mice were backcrossed to C57BL/6 mice 5 times. Heterozygotes were then interbred to obtain homozygotes. Genotyping was performed using polymerase chain reaction (PCR) and confirmed with Southern blotting using tail genomic DNA as a template. Southern blotting was performed using probe-labeling with the Rediprime II DNA labeling kit (GE Healthcare, Little Chalfont, UK) and imaged with BAS-1500 (Fuji). For mouse genotyping by PCR, *Snx5*-specific forward and reverse primers were designed (common forward primer: GGAGATGTTTGGAGGCTTTTT, wild-type reverse primer: CAGGAATCCTTGATCCTGTTG, mutant reverse primer: GGTCGAGAGAACGGTGAGAG). Genomic DNA samples were digested with *Eco*RI and probed with a 5′external probe.

### Isolation and culture of primary mouse lung epithelial cells

Lung tissues from *Snx5^+/-^* intercrosses were collected by caesarean section at E18.5. Primary lung epithelial cells were cultured with the methods described by Warshamana and Matsui [32], [33] with slight modifications, and were used for EGF treatment experiments. Briefly, isolated lungs were removed and incubated with dispase (5 mg/mL, *STEMCELL* Technologies, Inc.) for 45 min at room temperature. The lungs were minced and transferred to Dulbecco’s modified Eagle’s medium (DMEM) with 0.01% DNase I (1 mg/mL, *STEMCELL* Technologies, Inc.) for 10 min at 37°C. The dissociated cells were filtered through a 40-µm nylon cell strainer and centrifuged at 1000 rpm for 10 min. The resuspended cells were plated into cell culture dishes at 37°C for 1 h in order to remove the macrophages and fibroblasts. After a 1-h incubation, nonattached cells were transferred into new cell culture dishes. This step was repeated 4 times. Final nonattached cells were filtered through a 40-µm strainer and centrifuged. The resuspended cells were plated on type-I collagen-coated 35 mm culture dishes (BD BioCoat) in DMEM/Ham’s F-12 50/50 mixed medium (Cellgro) supplemented with 15 mM HEPES, 0.8 mM CaCl_2_, 0.25% BSA, 5 µg/mL insulin, 5 µg/mL transferrin, 5 ng/mL sodium selenite, and 2% mouse serum. After 24 h, the culture medium was changed to serum-free DMEM/Ham’s F-12 mixed medium supplemented as described above and maintained in the same medium. The isolated cells were almost all type II cells, as determined by qRT-PCR and immunocytochemistry analysis of T1α− and pro-SP-C.

### Cell culture, EGF stimulation, and CHX treatment

Primary MEFs were isolated from embryonic day 13.5 (E13.5) *Snx5^+/+^* and *Snx5^-/-^* embryos. MEFs were maintained in Dulbecco’s modified Eagle’s medium (DMEM; Hyclone) containing 10% fetal bovine serum (FBS; Hyclone), antibiotic-antimycotic solution (Invitrogen, Carlsbad, CA, USA), and 2-mercaptoethanol (Invitrogen) in a humid atmosphere of 5% CO_2_ at 37°C. For EGF treatments, MEFs were serum-starved for 12 h and then treated with 100 ng/mL EGF for 0, 5, 10, 20, 60, 120 and 180 min and 0, 5, 15, 30, 60 and 120 min. For CI-MPR protein detection, cells were treated with 40 µg/mL cycloheximide (CHX; Sigma, St. Louis, MO, USA) for 17 h to inhibit biosynthesis of CI-MPR protein.

### RNA isolation and quantitative RT-PCR analysis

Total RNA was isolated from lung tissues using TRIzol Reagent (Invitrogen, Carlsbad, CA) and complementary DNA synthesis was performed according to the manufacturer’s instructions (ImProm-II Reverse Transcription system; Promega). Quantification of cDNAs from specific mRNA transcripts was accomplished by qRT-PCR (Bio-Rad) using SYBR Green PCR mixture (Takara Bio Inc.) as described previously [25]. The relative concentration of each mRNA was normalized to the concentration of 18S rRNA in each sample. PCR primer sequences are shown in Table S2.

### Western blot analysis and measurement of saturated phosphatidylcholine (Sat PC)

Protein detection in murine embryonic fibroblasts (MEFs), primary lung epithelial cells (PLEs) and total lung lysates of *Snx5^+/+^* and *Snx5^-/-^* mice by western blot has been described previously [27]. Proteins were detected using specific primary antibodies at 1∶1000 dilution, including rabbit anti-epidermal growth factor receptor (EGFR), anti-cation-independent mannose 6-phosphate receptor (CI-MPR), anti-phospho-Akt, anti-Akt, anti-phospho-Erk1/2, anti-Erk1/2 (Cell Signaling Technology, Danvers, MA, USA), rabbit anti-tubulin (Abcam), rabbit anti-β-actin (Sigma), rabbit anti-Snx5, goat anti-CC-10, mouse anti-PCNA, goat anti-Pecam-1 (Santa Cruz Biotechnology, Santa Cruz, CA, USA), rabbit anti-proSP-C (Chemicon), Syrian hamster anti-T1α (1:100, Developmental Studies Hybridoma Bank), rabbit anti-pro-SP-C (1:2000, Chemicon), goat anti-CC-10 (1:2000, Santa Cruz Biotechnology), mouse anti-α-SMA (1:500, Santa Cruz Biotechnology), mouse anti-SP-B (Seven Hills Bioreagents, Cincinnati, OH, USA), mouse anti-vimentin (1:1000, Chemicon), and mouse anti-E-cadherin (1:1000, BD Biosciences). The membranes were incubated with horseradish peroxidase-conjugated secondary antibodies (goat anti-rabbit IgG, goat anti-mouse IgG, donkey anti-goat IgG or goat anti-Syrian hamster IgG, as appropriate; Promega or Santa Cruz Biotechnology). Protein bands were detected using ECL Reagent (Thermo) followed by exposure to X-ray film (Agfa) or detection in a Vilber Fusion Solo 2 chemiluminescence system (Fisher Biotec). SP-B immunoblot bands were quantified by densitometry (ImageQuant v5.2, GE Healthcare, Piscataway, NJ). Sat PC was isolated from lipid extracts of lung homogenate by using osmium tetroxide followed by phosphorus assay as described previously [34], [35], [36].

### Histology and β-galactosidase staining

Embryonic lungs were dissected and fixed in 4% paraformaldehyde (PFA) for 24 h at 4°C. The lungs were dehydrated in a series of graded ethanol solutions and embedded in paraffin blocks. Histological analysis was performed on 5-µm sections. Hematoxylin and eosin (H&E), periodic acid Schiff (PAS), and Alcian blue staining were performed according to standard protocols [37]. For β-galactosidase staining, whole lungs were fixed in 4% PFA (pH 7.4) for 2 h at 4°C. The tissues were washed in phosphate-buffered saline (PBS) and placed in X-gal staining solution (1 M MgCl_2_, 0.01% sodium-deoxycholate, 0.02% NP-40, 5 mM potassium ferricyanide/ferrocyanide, and 1 mg/mL X-gal in PBS) overnight at 37°C.

### Floating assay

Floating assay was performed as previously described [15], [38], [39], [40]. Briefly, as soon as *Snx5^-/-^* mice had died, the lungs were removed from newborn mice and deposited in eppendorf tubes containing PBS.

### Air-breathing test

Mice were collected by caesarean section at E18.5. We cleaned the bodies and immediately removed the lung fluid. To maintain body temperature, the mice were incubated in a 37°C chamber. The mice were exposed to room air. Most mice exhibited redness and normal breathing. At this time, we set the time to zero and then observed the phenotypes of these pups for 100 min. *Snx5^+/+^* and *Snx5^+/-^* mice showed less than 10% mortality over the duration of the study, exhibiting normal breathing through the mouth.

### Immunohistochemistry and immunocytochemistry

Sections were deparaffinized, rehydrated, and processed for antigen retrieval with 0.01 M citric acid solution (pH 6.0) for 15 min. Sections were then incubated with 3% H_2_O_2_ in methanol for 15 min to quench endogenous peroxidase activity. After blocking with blocking solution (5% horse serum, 3% bovine serum albumin, 0.1% Triton X-100 in PBS), the slides were incubated with primary antibodies in blocking solution overnight at 4°C. Antibodies used for immunohistochemistry were as follows: hamster anti-T1α (1∶100, Developmental Studies Hybridoma Bank), rabbit anti-pro-SP-C (1:2000, Chemicon), goat anti-CC-10 (1∶2000, Santa Cruz Biotechnology), mouse anti-α-SMA (1:500, Santa Cruz Biotechnology), rabbit anti-Ki67 (1∶500, Novo Castra), and goat anti-Pecam-1 (1∶100, Santa Cruz Biotechnology). The slides were washed with PBS and then incubated with Alexa 594 goat anti-rabbit IgG at a 1∶200 dilution, Alexa 488 goat anti-rabbit IgG at a 1∶200 dilution, Alexa 488 goat anti-mouse IgG at a 1∶200 dilution, Alexa 594 goat anti-mouse IgG at a 1∶200 dilution, Alexa 594 donkey anti-goat IgG at a 1∶200 dilution, Alexa 488 goat anti-hamster IgG at a 1∶200 dilution (Invitrogen), or reagents from a Vectastain Elite ABC kit (Vector Laboratories) for secondary detection. Immunocytochemistry was performed in *Snx5* MEF cell lines and PLEs. MEFs on gelatin-coated cover glass and PLEs on type I-coated cover glass were fixed in 4% PFA for 15 min on ice and then washed with 0.1% Triton X-100 in PBS. The samples were incubated with primary rabbit anti CI-MPR (1∶100, Abcam), hamster anti-T1α (1∶100, Developmental Studies Hybridoma Bank), or rabbit anti-pro-SP-C (1∶2000, Chemicon) antibodies for 2 h at room temperature followed by incubation with secondary antibodies, i.e., AlexaFluor 594 goat anti-rabbit IgG (1∶200, Invitrogen) and Alexa 488 goat anti-hamster IgG, for 1 h at room temperature. The cover glasses were mounted with Vectashield Mounting Medium with DAPI (VECTOR, H-1200). Images were obtained using an Axio Imager A2 microscope (Carl Zeiss, Germany) and Olympus software (Olympus micro DP71/DP70/DP30 BW Ver.03.03).

### Transmission electron microscopy (TEM)

To examine the ultrastructure of E18.5 lung morphology, E18.5 lungs were collected and fixed in modified Karnovsky’s fixative overnight at 4°C. Fixed tissues were washed 3 times with 0.05 M sodium cacodylate buffer (pH 7.2) and postfixed with 1% osmium tetroxide in 0.05 M sodium cacodylate buffer for 2 h. Tissues were dehydrated in graded ethanol solutions and propylene oxide and then embedded in Spurr’s resin. Ultrathin sections were stained with uranyl acetate and Reynold’s lead citrate. The sections were imaged with a LIBRA 120 TEM (Carl Zeiss, Germany).

### Microarray analysis

Expression profiles of *Snx5^+/+^* and *Snx5^-/-^* in E18.5 mouse lungs were generated using an Illumina microarray system (Illumine Beadchip Array MouseRef-8 v2), which includes 25,697 probes corresponding to 17,214 annotated genes. Total RNA was isolated from *Snx5^+/+^* and *Snx5^-/-^* lungs using the RNeasy MiniKit (Qiagen). RNA integrity numbers (RIN) ranged from 9.3 to 9.7 when analyzed using an Agilent 2100 Bioanalyzer. RNA was reverse transcribed and amplified using Illumina Total Prep RNA amplification kit (Ambion). *In vitro* transcription was then carried out to prepare cRNA. The cRNAs were hybridized to the array and then labeled with Cy3-streptavidin (Amersham Bioscience). The fluorescent signal on the array was measured using a BeadStation 500 System (Illumina). In this study, two biological replicates of *Snx5^+/+^* and *Snx5^-/-^* lungs were analyzed. The microarray data used in this study were deposited in the GEO database (GSE37080).

### Statistical analysis of gene expression data

The probe intensities from the arrays were converted to log2 intensities. The log2 intensities were then normalized using a quantile normalization method [41]. The probes were annotated using (GPL6885.annot). A gene was considered “expressed” if the probe intensity of the gene was larger than a cutoff intensity in at least one condition. The cutoff intensity (in this study, intensity  =  8) was determined by mixture modeling using 2 Gaussian distributions, one for the “absent” probes and the one for the “present” (expressed) probes [42] . To identify differentially-expressed genes (DEGs) by comparing *Snx5^-/-^* vs. *Snx5^+/+^* in E18.5 lungs, the following integrative statistical hypothesis-testing was performed: (1) Student *t*-test and log2 median ratio test were performed to compute *T*-values and median ratios for all the genes [42], [43]; (2) *P*-values from each test were computed using an empirical distribution of the null hypothesis (that the means of the genes were not different), which was obtained from random permutations of the samples; (3) the individual *P*-values were combined to compute the false discovery rate (FDR) using Stouffer’s method [43]; and (4) the DEGs were selected as the genes with an FDR of less than 0.05 and log2-fold change larger than the cutoff of 0.3531 (1.27-fold change at the original scale). This fold-change cutoff value corresponded to the 95^th^ percentile of the fold changes obtained from random permutation experiments using 2-tailed settings. Finally, functional enrichment analysis of DEGs was performed using DAVID software to identify cellular processes overrepresented by the DEGs governed by *Snx5*.

### Statistics

In this study, all quantitative graphs are expressed as means ± standard deviations (SDs). *P*-values were calculated by Student’s *t*-tests. A *P-*values ≤ 0.05 was considered statistically significant.

## Results

### Generation of *Snx5^-/-^* mice and expression of *Snx5*

**Figure 1:**
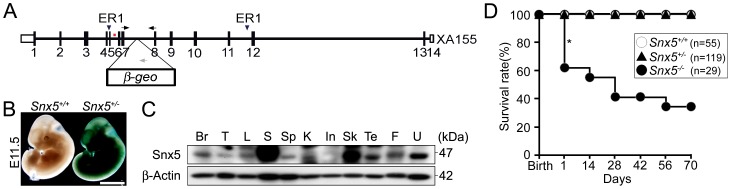
Generation of *Snx5*-deficient mice. (A) Schematic drawing of the *Snx5* gene-trapped (XA155) allele. Arrows indicate the genotyping primers, and the 2 arrowheads indicate the ER1 (*Eco*RI) digestion sites. The red bar represents the probe for Southern blotting. (B) Whole-embryo X-gal staining at E11.5. Scale bars: 2.5 mm. (C) Western blot analysis of the indicated mouse tissues (Br, brain; T, thymus; L, lung; S, stomach; Sp, spleen; K, kidney; In, intestine; Sk. skin; Te, testis; F, fat; U, uterus). (D) Survival rate of mice after birth. The asterisk shows the dramatic mortality observed in the neonatal stage.

To determine the physiological role of *Snx5*, we generated *Snx5* heterozygous mice (*Snx5^+/-^*) using the gene-trapped ES cell line XA155, containing a β-galactosidase-neomycin cassette (β-geo) between exons 7 and 8 (Figure 1A). Germline transmission of the trapped allele was confirmed by Southern blot analysis with an *Snx5*-specific probe (Figure S1A). Inactivation of the *Snx5* gene was confirmed by genomic DNA PCR (Figure S1B), RT-PCR (Figure S1C), and western blotting (Figure S1D). Through β-galactosidase staining of *Snx5^+/-^* mice expressing the β-galactosidase gene, we examined the expression patterns of *Snx5*. β-galactosidase staining of E11.5 *Snx5^+/-^* tissues (Figure 1B) and adult tissues (data not shown) revealed ubiquitous expression of *Snx5*. Western blot analysis of adult tissues from *Snx5^+/+^* mice also showed that *Snx5* was expressed in various adult tissues (Figure 1C), suggesting that *Snx5* may play a role in embryonic and adult developmental stages in various tissues.

### Perinatal lethality and respiratory failure in *Snx5^-/-^* mice

Examination of the survivability of *Snx5^-/-^* mice showed that 40% of mutant mice died at birth. Until E17.5, embryos from *Snx5^+/-^* intercrosses showed the expected Mendelian ratio. However, at E18.5, *Snx5^-/-^* embryos were born at a frequency of 19.4%, lower than the expected Mendelian ratio of 25% (Table 1).

**Table 1:** Embryonic survival frequency of *Snx5^+/+^*, *Snx5^+/-^* and *Snx5^-/-^* littermates.

Genotype n (%)			
**Age of embryos (day)**	***Snx5^+/+^***	***Snx5^+/-^***	***Snx5^-/-^***	***Total n***
**E14.5 ∼ E16.5**	33 (20.3)	88(54.3)	41(25.3)	162
**E17.5**	18(23.3)	38(49.3)	21(27.2)	77
**E18.5**	124(30.2)	207(50.3)	80(19.4)*	411

Embryos from timed matings were genotyped on embryonic day 14.5∼16.5, 17.5 and 18.5 day. Fourth columm shows the observed percentage of Snx5-/- mice (the Mendelian percentage is 25%).

* Caculated using *χ^2^* test comparing the expected and observed frequency of viable *Snx5^-/-^* mice. *(P = 0.0089)*

**Figure 2:**
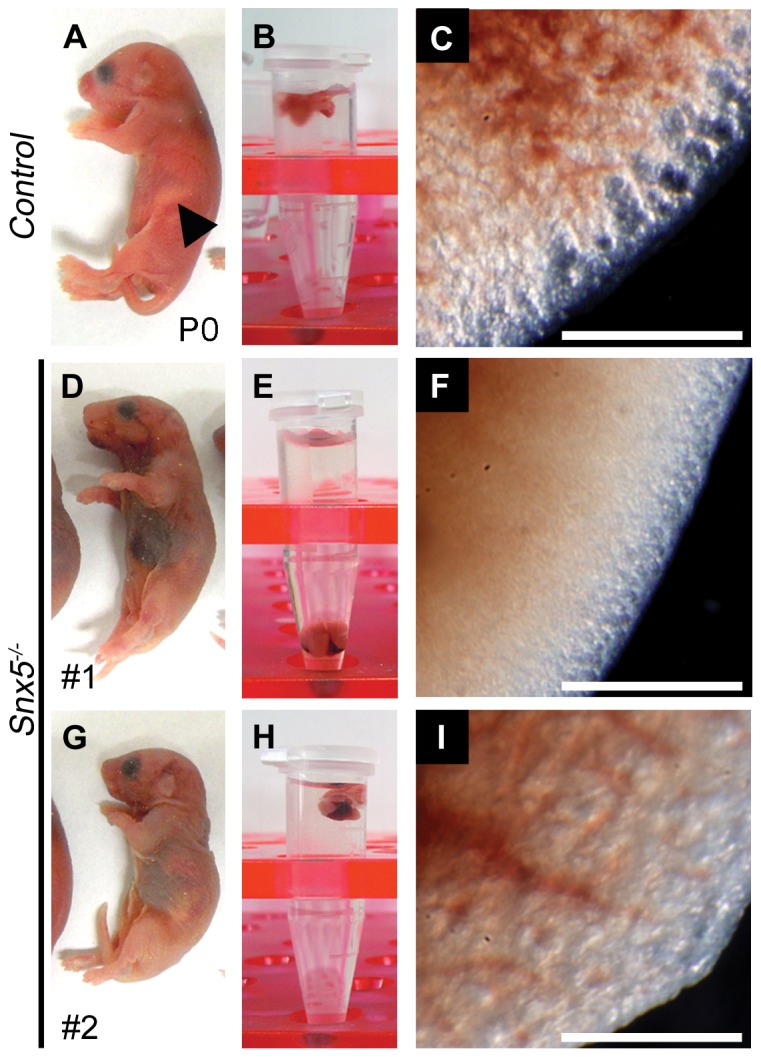
Perinatal lethality in *Snx5^-/-^* mice. (A, D, G) Appearance of neonates after birth. The arrowhead indicates milk in the stomach. (B, E, H) Floating assay of the lungs. (C, F, I) Appearance of the lungs. Control lungs show the inflated morphology (C). *Snx5^-/-^* lungs lack inflated morphology (F) or show partially inflated morphology (I). Scale bars: 0.5 mm.

Interestingly, 40% of *Snx5^-/-^* mice died within 24 h of birth (Figures 1D and 2D) and an additional 20% of *Snx5^-/-^* mice died within a month of birth (Figure 1D). The surviving *Snx5^-/-^* mice had a smaller body size compared to that of *Snx5^+/-^* and *Snx5^+/+^*mice after birth (Figure S1E and F). Newborn *Snx5^+/+^* and *Snx5^+/-^* mice were pink and exhibited a normal suckling reflex and well-inflated lungs (Figure 2A–C). However, most *Snx5^-/-^* mice were cyanotic (Figure 2D and G) and had condensed lungs (Figure 2E and F), although some *Snx5^-/-^* lungs were partially inflated with air at birth (Figure 2H and I). These results suggested that *Snx5^-/-^* mice may experience respiratory failure at birth.

In order to investigate the causes of respiratory failure in *Snx5^-/-^* mice, we examined the palate, trachea, esophagus, diaphragm, intercostal muscles, and heart. In *Snx5^-/-^* mice, the palate was closed well (Figure S2A and B), and the structures of the trachea and esophagus were formed and separated properly (Figure 2C–F and 2K and L). The structure of the tracheal cartilage ring was also properly formed in mutant mice (Figure S2M and N). Histological analysis of other respiratory organs, such as the diaphragm (Figure S2G and H) and intercostal muscles (Figure S2I and J) of *Snx5^-/-^* mice showed well-formed morphologies. In addition, the heart showed the normal morphological structure in *Snx5^-/-^* mice (Figure S2O and P). Collectively, these results suggested that the respiratory failure of *Snx5^-/-^* mice was not due to malformations of the palate, trachea, esophagus, diaphragm, intercostal muscles, or heart.

### Impaired alveolar formation in *Snx5^-/-^* mice

**Figure 3:**
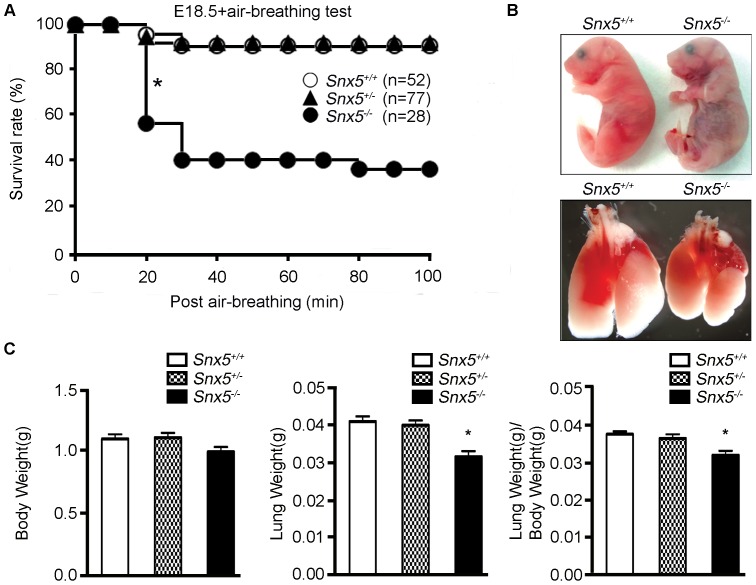
Respiratory failure in *Snx5^-/-^* mice. (A) Survival after air-breathing tests in *Snx5^+/+^*, *Snx5^+/-^*, and *Snx5^-/-^* mice. Within 30 min, nearly 60% of *Snx5^-/-^* mice had died (asterisk). (B) Appearance after air-breathing tests in E18.5 mice. Cyanotic appearance and condensed lungs in *Snx5^-/-^* mice. (C) Body and lung weights of *Snx5^+/+^*, *Snx5^+/-^*, and *Snx5^-/-^* mice. Lung weight and lung to body weight ratios of *Snx5^-/-^* mice were decreased by 22.9% and 14.9%, respectively, relative to *Snx5^+/+^* mice. *P < 0.0001; data represent *Snx5^+/+^*, n  =  24; *Snx5^+/-^*, n  =  27; and *Snx5^-/-^* mice, n  =  17.

Considering that the structures of palate, trachea, esophagus, diaphragm, intercostal muscles, and heart were intact, we hypothesized that the respiratory failure observed in these mice could be due to defects in lung development. To test this possibility, we performed an air-breathing test after caesarean section at E18.5. After removing the pups from their mother, we measured the time until death from the point the pup began normal breathing in a 37°C chamber, intended to help the mouse maintain body temperature. While *Snx5^+/+^* and *Snx5^+/-^* mice survived after beginning to breathe, approximately 60% of mutant mice died within 1 h (Figure 3A and B). Moreover, while the body weights of *Snx5^-/-^* mice was similar to those of control mice at E18.5, lung weights and lung-to-body weight ratios of *Snx5^-/-^* mice were slightly lower than those of *Snx5^+/+^* and *Snx5^+/-^* mice (Figure 3C). Together with the results of the air-breathing test, these data suggested that lung defects in *Snx5^-/-^* mice could lead to the observed high mortality rate at birth.

**Figure 4:**
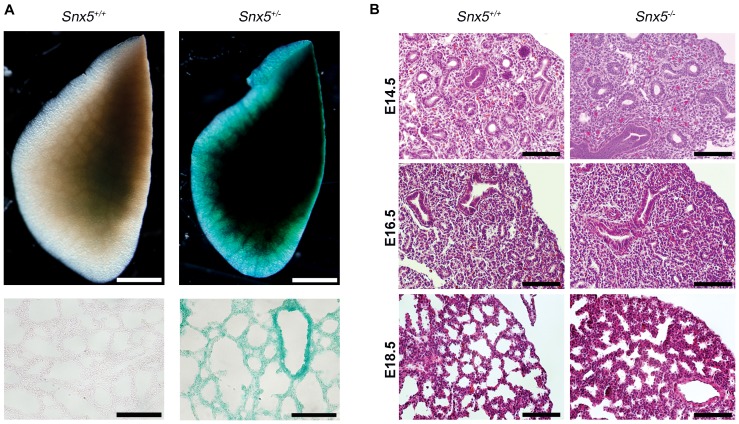
Expression of *Snx5* and morphological malformations in *Snx5*-deficient mice at E18.5. (A) Whole and sectional X-gal staining of E18.5 lungs. The *Snx5- βgeo* reporter gene was expressed ubiquitously in *Snx5^+/-^* lungs. Scale bars: 1 mm, 100 µm. (B) H&E staining of E14.5, E16.5, and E18.5 *Snx5^+/+^* and *Snx5^-/-^* lungs. *Snx5^-/-^* lungs exhibited thickening of the alveolar walls and reduced alveolar space at E18.5. Scale bar: 50 µm.

Next, in order to investigate whether *Snx5* was expressed in the lung, we performed β-galactosidase staining on E18.5 *Snx5^+/-^* lungs. As expected, *Snx5* was expressed throughout the lung (Figure 4A), suggesting that *Snx5* may play a role in lung development. In addition, hematoxylin and eosin staining revealed that *Snx5^-/-^* lungs had thickened alveolar walls and reduced air space in the alveolar sacs at E18.5. Alveolar walls and air space were normal in E14.5 and E16.5 lungs (Figure 4B), suggesting that *Snx5* was required for alveolar formation in the saccular phase of lung development.

**Figure 5:**
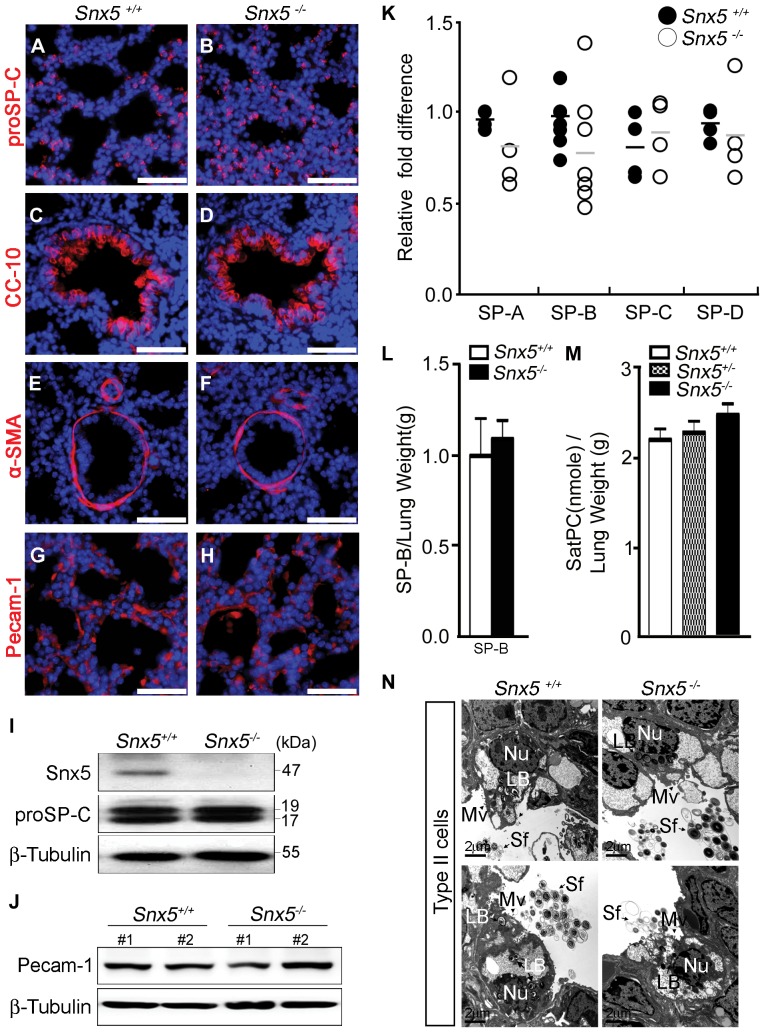
*Snx5* was not required for maturation of alveolar epithelial type II cells. (A–H) Immunohistochemical analysis of proSP-C, CC-10, α-SMA, and Pecam-1 in sections from *Snx5^+/+^* and *Snx5^-/-^* lungs (n  =  5). Scale bars: 50 µm. (A and B) proSP-C, a marker of surfactant protein C precursor in alveolar epithelial type II cells; (C and D) CC-10, a marker of Clara cells; (E and F) α-SMA, a marker of smooth muscle cells; and (G and H) Pecam-1, a marker of blood vessels, showed similar expression levels. The nuclei were stained with Hoechst. (I and J) Western blot analysis of proSP-C (I, n  =  5) and Pecam-1 (J, n  =  2) proteins in E18.5 *Snx5^+/+^* and *Snx5^-/-^* lung whole lysates. (K) qRT-PCR analysis of surfactant proteins (SP-A, -B, -C, and -D) performed on RNA samples collected from total lungs (n ≥ 3). Similar expression levels of surfactants were seen. (L) Mature SP-B did not differ in *Snx5^-/-^* lungs (n  =  5). (M) Sat PC were similar between *Snx5^+/+^*, *Snx5^+/-^*, and *Snx5^-/-^* mice (n  =  6). (N) Transmission electron microscopy (TEM) of distal alveolar epithelial type II cells at E18.5. Apical microvilli (Mv; arrowhead), condensed lamellar bodies (LB), and secreted surfactants (Sf; arrow) were observed in *Snx5^-/-^* lungs. Scale bars: 2 µm.

To examine whether the observed impaired alveolar formation was due to defective differentiation of pulmonary epithelial cells or defective vascular formation, we performed immunohistochemistry with specific antibodies against alveolar epithelial type II cells (proSP-C), Clara cells (CC-10), smooth muscle cells (α-SMA), and blood vessels (Pecam-1). In E18.5 *Snx5^-/-^* mice, proSP-C-expressing cells were similar to those of controls (Figure 5A and B). Western blot analysis also showed similar levels of proSP-C in *Snx5^-/-^* lungs (Figure 5I). The expression of CC-10 in mutant bronchioles was comparable to that of *Snx^+/+^* mice (Figure 5C and D), suggesting that proximal lung development was intact in mutant mice. In addition, immunohistochemical staining of α-SMA and Pecam-1 did not show significant differences between *Snx5^+/+^* and *Snx5^-/-^* lungs (Figure 5E–H and J), indicating that the terminal vascular architecture was intact in the mutant lungs. Furthermore, we also investigated whether thickened alveolar septa were correlated with increased proliferative cells in *Snx5^-/-^* lungs. At E18.5, Ki67 staining of the *Snx5^-/-^* lungs showed similar amounts of proliferating cells relative to controls (Figure S3A and B), indicating that the thickened alveolar septa of *Snx5^-/-^* lungs were not caused by increased proliferation of epithelial cells.

Most lung atelectasis is caused by defective maturation of alveolar epithelial type II cells [44]. Mature alveolar epithelial type II cells secrete surfactants that reduce pulmonary surface tension and prevent atelectasis at the end of expiration [45]. These surfactants are composed of a complex mixture of proteins and lipids. To investigate whether the impaired alveolar formation was due to a maturation defect of alveolar epithelial type II cells, we compared the expression levels of surfactant proteins and lipids in *Snx5^+/+^* and *Snx5^-/-^* lungs. Alveolar epithelial type II cells expressed the surfactant proteins SP-A, -B, -C, and -D. qRT-PCR analysis showed that the mRNA expression levels of these surfactant proteins were not significantly different between *Snx5^+/+^* and *Snx5^-/-^* lungs (Figure 5K). The amount of SP-B in 15 μL of supernatant from lung homogenates (total volume 2 mL) was analyzed by western blotting (Figure S4A and B). SP-B protein/lung weight ratios were not altered by deletion of *Snx5* (Figure 5L). Levels of Sat PC, the most abundant component of surfactant lipids, were slightly lower in *Snx5^-/-^* lungs than in *Snx5^+/-^* lungs (Figure S4C). However, Sat PC content relative to lung weight was similar between groups (Figure 5M). Likewise, ultrastructure analysis by TEM revealed that *Snx5^-/-^* lungs had normal alveolar epithelial type II cells with compacted, well-organized lamellar bodies and had secreted surfactants in the alveolar air-space (Figure 5N). Taken together, it can be concluded that deletion of *Snx5* does not appear to affect the maturation of alveolar epithelial type II cells.

### Impaired differentiation of alveolar epithelial type I cells in *Snx5^-/-^* lungs

**Figure 6:**
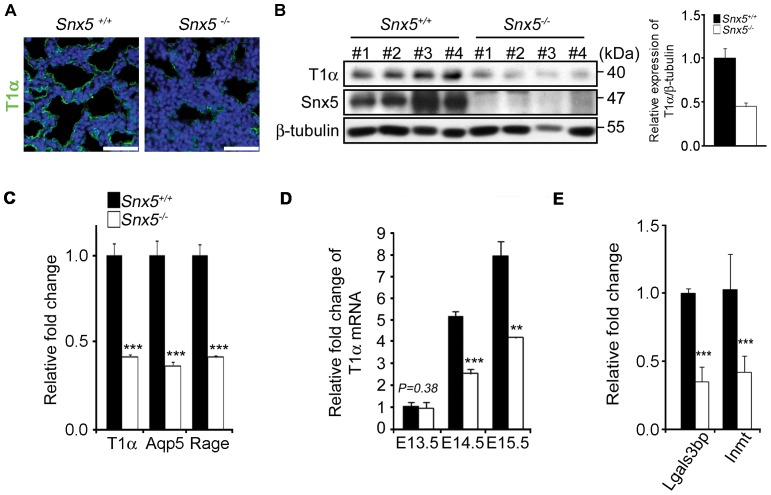
Impaired differentiation of alveolar epithelial type I cells in E18.5 *Snx5^-/-^* lungs. (A) Immunohistochemical analysis of T1α at E18.5. T1α, a marker of alveolar epithelial type I cells, was significantly decreased in *Snx5^-/-^* compared to *Snx5^+/+^* lung (n  =  5). The nuclei were stained with Hoechst. Scale bars: 50 µm. (B) Decreased protein expression of T1α in E18.5 (n  =  4). Densitometric analysis showed that average expression levels of T1α in *Snx5^-/-^* lungs were reduced compared to those in *Snx5^+/+^* lungs. (C–E) qRT-PCR analysis of type I cell-related genes from lung tissue. (C) Markers of alveolar epithelial type I cells, i.e., *T1α*, *Aqp5*, and *Rage*, were decreased in *Snx5^-/-^* lungs (n ≥ 3). (D) *T1α* mRNA during the embryonic stage. From E14.5, mRNA expression of *T1α* was decreased. (E) Decreased expression levels of *Lgals3bp* and *Inmt* (n ≥ 3). **, *P* < 0.01, ***, *P* < 0.001 using the Student’s *t*-test.

In order to further investigate the causes of respiratory failure in the *Snx5^-/-^* mice, we examined whether the differentiation of alveolar epithelial type I cells was intact. Immunohistochemical analysis of T1α, a marker of typical alveolar epithelial type I cells, revealed that the expression of T1α was markedly decreased in *Snx5^-/-^* lungs, whereas T1α expression extended along the alveolar wall in control lungs (Figure 6A). Consistently, the expression levels of T1α in lung lysates from E18.5 *Snx5^-/-^* mice were dramatically decreased compared to those of controls (Figure 6B). Additionally, the typical markers for distal alveolar epithelial type I cells, such as T1α, AQP5, and RAGE were examined [46]. Indeed, the mRNA expression levels of *T1*α, *Aqp5*, and *Rage* were significantly decreased in E18.5 *Snx5^-/-^* lungs compared to those of controls (Figure 6C). Thus, we hypothesize that loss of *Snx5* leads to impaired differentiation of alveolar epithelial type I cells.

Reduced mRNA expression of T1α in *Snx5^-/-^* lungs was evident from E14.5 (Figure 6D). Next, we examined the expression levels of lectin, galactoside-binding, soluble, 3 binding protein (Lgals3bp) and indolethylamine *N*-methyltransferase (Inmt), which have been shown to be reduced in a defective type I epithelial cell-associated mouse model, SMRT^mRID^ mice [15]. Pei and colleagues reported that differentiation-defect of type I pneumocytes promoted respiratory distress syndrome at perinatal stages. In this paper, genome-wide analysis of mutant mice showed that several genes involved in Lgals3bp and Inmt signaling were reduced in mutant mice. To determine whether decreased T1α affected the expression of these genes, we performed qRT-PCR. We observed that *Lgals3bp* and *Inmt* transcripts were significantly reduced in *Snx5^-/-^* lungs (Figure 6E). Next, to further investigate the morphological differentiation of alveolar epithelial type I cells, we performed the ultrastructure analysis by using TEM. Although *Snx5^-/-^* lungs showed a less alveolar epithelial type I cells compared to *Snx5^+/+^* lungs (data not shown), The alveolar epithelial type I cells of *Snx5^-/-^* lungs represented slightly thicker nucleus and cytoplasm (Figure S5B and D) compared to *Snx5^+/+^* lungs (Figure S5A and C). However organelles enriched in alveolar epithelial type I cells, such as mitochondria (Figure S5A and B ; white arrowhead) and vesicles (Figure S5A and B ; black arrow), existed well in *Snx5^-/-^* lungs (Figure S5B). Also, extended cytoplasms were observed in both groups (Figure S5C and D). It seems that there were no obvious differences in ultrastructural morphologies of alveolar epithelial type I cells. From these data, we concluded that *Snx5* was required for proper differentiation of alveolar epithelial type I cells.

### *Snx5* as a novel mediator required for differentiation of alveolar epithelial type I cells

**Figure 7:**
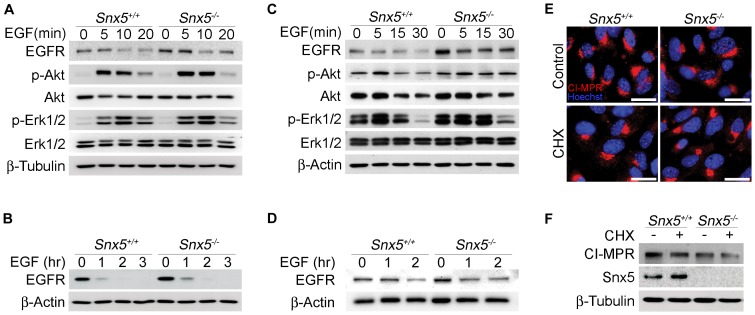
Regulation of EGFR signaling and CI-MPR trafficking were not altered in *Snx5*-deficient MEFs. (A–D, F) Western blotting of EGFR and CI-MPR. (A and C) Signaling downstream of EGFR and (B and D) degradation of EGFR were not altered in primary *Snx5^-/-^* MEFs (A and B) and primary lung epithelial cells (PLEs) (C and D). (E) Immunocytochemical analysis of CI-MPR. Localization of CI-MPR was not changed in *Snx5*-deficient MEFs. (F) Relative degradation ratios of CI-MPR protein in *Snx5^+/+^* and *Snx5^-/-^* MEFs.

*Snx5* has been implicated in endosomal trafficking of EGFR and CI-MPR [18], [19], [20], [47]. In order to investigate whether impaired differentiation of alveolar epithelial type I cells in *Snx5^-/-^* lungs was due to defective endosomal trafficking of EGFR, we examined EGFR signaling after EGF stimulation in *Snx5^-/-^* MEFs and isolated PLEs (Figure S6A-C). Expression levels of EGFR were comparable between *Snx5^+/+^* and *Snx5^-/-^* MEFs (data not shown). To further investigate the EGFR signaling, the downstream signaling molecules were measured by using immunoblot in EGF stimulated *Snx5^+/+^* and *Snx5^-/-^* MEFs. Phosphorylation of Akt and Erk1/2, the key effectors of EGFR signaling, was comparable between *Snx5^+/+^* and *Snx5^-/-^* MEFs in response to EGF treatment (Figure 7A and C). In addition, after long-term exposure to EGF, there was no difference in the downregulation of EGFR (Figure 7B and D). The *Snx5* deletion did not affect the endosomal signaling and degradation of EGFR in MEFs and PLEs and the expression levels of EGFR in the E18.5 *Snx5^-/-^* lungs were also similar between two groups (Figure S7A and B).

Next, we examined whether endosome-to-TGN retrieval of CI-MPR was affected by disruption of *Snx5*. CI-MPR has been reported to be a major retromer component in retrograde endosome-to-TGN transport [24]. Knockdown of *Snx5* using small interfering RNA inhibits trafficking of CI-MPR from endosomes to the TGN, resulting in increased degradation of CI-MPR [20], [48]. To establish whether the local alteration of CI-MPR resulted from Snx5 depletion, we performed immunocytochemistry analysis of CI-MPR (Figure 7E). Additionally, to determine CI-MPR stability in *Snx5^-/-^* MEFs, we examined the degradation rate of CI-MPR (Figure 7F). Because the biosynthetic rate of CI-MPR protein varies in efficiency, we treated cells with 40 µg/mL CHX for 17 h to compare the degradation ratio of CI-MPR in *Snx5^+/+^* and *Snx5^-/-^* MEFs. Unexpectedly, however, when we examined the localization and stability of CI-MPR in *Snx5^-/-^* MEFs, there was no significant alteration in *Snx5^-/-^* MEFs compared to *Snx5^+/+^* MEFs (Figure 7E and F). This finding suggests that the loss of *Snx5* alone in a retromer complex does not affect the localization and lysosomal degradation of CI-MPR in MEFs.

No studies have reported the correlation between Snx5 and the differentiation of alveolar epithelial type I cells. Since there was no clear molecular explanation for the defective differentiation of alveolar epithelial type I cells in *Snx5^-/-^* mice, a genome-wide cRNA microarray analysis using the RNA isolated from E18.5 *Snx5^+/+^* and *Snx5^-/-^* lungs was performed. Microarray analysis data using an Agilent 2100 Bioanalyzer [49] revealed the decreased expression of more than 60 genes in *Snx5^-/-^* lungs compared to *Snx5^+/+^* lungs (data not shown). The altered genes were members of several functional categories, including genes for pulmonary epithelial cell markers, development, maturation, regulation of endocytosis and transport, serine/threonine kinases, enzyme-linked receptor signaling pathways, cell adhesion, and cell migration. Among these downregulated genes, we found that expression of the alveolar epithelial type I cell markers *T1α*, *Aqp5*, and *Rage* was significantly decreased (data not shown). In addition, several genes involved in ribosome biogenesis, RNA processing, translation and oxidative phosphorylation exhibited upregulated expression (Table S1). However, from microarray analysis data and qRT-PCR, we concluded that there was no significant difference in Notch signaling-related genes, such as *Hes1*, *Hes5*, *Hey1*, *Hey2*, and *HeyL*, between groups (data not shown). Thus, these data raise the possibility that *Snx5* may be involved in other signaling pathways in addition to the Mib1-Notch signaling pathway. To address this question, we performed a literature search for previous studies on Snx5. Recently, Pei et al. reported that defective differentiation of alveolar epithelial type I cells led to a respiratory distress syndrome-like phenotype in SMRT mutant mice and that 21 genes, including *Klf2*, *Lgals3bp*, *Klf4*, *Inmt*, *Zfp36*, and *Dusp1*, were significantly decreased [15]. Interestingly, when we examined the expression levels of these genes in *Snx5^-/-^* lungs, we also found that these genes were significantly downregulated (data not shown). Taken together, although more molecular studies on Snx5 are required to determine the precise role of this protein in lung development, these finding suggest that *Snx5* could be a novel mediator for proper differentiation of alveolar epithelial type I cells.

## Discussion

Here, we report that *Snx5* is necessary for the differentiation of alveolar epithelial type I cells. Targeted inactivation of the *Snx5* gene led to neonatal mortality caused by respiratory failure, in which differentiation of alveolar epithelial type I cells was impaired, while maturation of alveolar epithelial type II cells was intact. Although SNX5 has been posited to interact with Mib-1, an E3 ubiquitin ligase that regulates the activation of Notch signaling, it is unlikely that these defects are caused by Mib1-Notch signaling. In addition, other related molecules, EGFR and CI-MPR, were not affected by Snx5 depletion. Collectively, our data reveal an essential role of Snx5 in the differentiation of alveolar epithelial type I cells.

Maturation defects of alveolar epithelial cells can cause neonatal respiratory failure. Thus far, various factors, such as C/EBPα, co-activator-associated arginine methyltransferase 1 (CARM1), Erk3, Foxa2, Foxm1, Fstl1, Mig6, and Pdpn (T1α), have been implicated in alveolar formation at the saccular stage of lung development. Disruption of these factors can lead to perinatal lethality caused by a respiratory distress syndrome (RDS)-like phenotype, which includes pulmonary atelectasis. This phenotype is largely caused by the excessive proliferation of immature alveolar epithelial type II cells [17], [35], [39], [50], [51], [52], [53]. In contrast, *Snx5^-/-^* lungs did not show a significant difference in cellular proliferation (Figure S3A and B), although cellularity was slightly increased. In addition, there were no significant differences between *Snx5^+/+^* lungs and *Snx5^-/-^* lungs in the production of surfactant proteins and Sat PC. Moreover, *Snx5^-/-^* lungs had normal alveolar epithelial type II cells with compacted, well-organized lamellar bodies and secreted surfactants in the alveolar air-space. This suggests that deletion of *Snx5* did not affect the maturation of alveolar epithelial type II cells and that the respiratory failure in the *Snx5^-/-^* mice was not due to functional defects of alveolar epithelial type II cells. However, *Snx5^-/-^* lung showed a significantly reduced expression of alveolar epithelial type I cell markers. Through these phenotypes, we could suggest that intact alveolar epithelial type II cells may reduce the severity of lung defects in *Snx5^-/-^* lung.

In present study, *Snx5^-/-^* mice showed the variability in survival rates. To determine whether leaky expression of *Snx5* could affect a different survival rates, we investigated the expression level of *Snx5* gene. In addition, we observed surviving and non-surviving *Snx5^-/-^* mice at birth and checked the expression levels of alveolar epithelial cell markers. Although we could not observed the differences between survivors and non-survivors, all mutant mice showed reduced expression of type I epithelial cell markers ; T1α, AQP5 and Rage (data not shown). So far, we did not define other causes of perinatal mortality in this study, we could suggest that the survivor mice have other complex compensatory mechanisms. Further studies will be required to address this possibility. In addition, the embryonic studies of *Snx5^-/-^* mice showed the significantly reduced numbers of mutant embryos at E18.5. Although the causes of embryonic death are ambiguous and need further studies, ubiquitous expressed of Snx5 suggests that Snx5 may play a role in various developmental contexts in embryonic stage.

Previous studies have demonstrated that EGFR-knockout mice exhibit growth retardation and epithelial immaturity, which shows partial similarity to the phenotype of *Snx5^-/-^* mice. *EGFR^-/-^* mice can be classified into 3 groups: runted, small-sized, and normal-sized pups [54]. The second group, small-sized pups, were shown to have breathing problems (gasping) and lived for up to 2 days. Dead *EGFR^-/-^* mice exhibited condensed and immature lung morphologies resembling neonatal respiratory distress syndrome. Although significant differences in EGFR signaling and degradation in MEFs after EGF stimulation were not observed in the current study, *Snx5^-/-^* and *EGFR^-/-^* mice display partially similar phenotypes such as lung immaturity, growth retardation and strain-dependent survivability. Although we did not go into details for postnatal phenotypes, we could observe that *Snx5^-/-^* mice had a longer lifespan and growth inhibition on other genetic background mice (data not shown). The inability to detect prominent differences in EGFR signaling and degradation in *Snx5^-/-^* MEFs may be due to the presence of a redundant signaling mechanism in MEFs and PLEs or the limited sensitivity of this experiment to detect subtle changes. Nevertheless, phenotypic similarities between *Snx5^-/-^* and *EGFR^-/-^* mice suggest that *Snx5* may be involved in EGFR signaling in the differentiation of alveolar epithelial type cells.

Knockdown of *Snx1* using RNA interference (RNAi) has been shown to cause dispersed cytosolic localization and degradation of CI-MPR in HeLa cells [22]. However, *Snx1/2*-deficient MEFs exhibit similar CI-MPR sublocalization and stability compared to wild-type MEFs [55], [56]. Similarly, although depletion of *Snx5* and *Snx6* in HeLa cells leads to dispersed CD8-CI-MPR trafficking into early endosomes [20], no significant differences in the localization and degradation of CI-MPR were observed in *Snx5^-/-^* MEFs in the current study. The lack of consensus in these findings suggests that the retrograde activity of CI-MPR may be regulated in a context-dependent manner in different cells and tissues.

We previously reported that *Snx5* interacts with Mib1, which plays a role in Notch signaling in various contexts of mammalian development [25], [26], [27], [28], [29], [30]. Snx5 morpholino injection into zebrafish embryos resulted in hematopoietic and blood vessel defects, which could be due to defective Notch signaling [21]. We hypothesized that *Snx5* could play an important role in Notch signaling and that *Snx5^-/-^* mice would exhibit Notch phenotypes; however, we did not observe Notch phenotypes, such as morphological defects of the somite, brain, vessels, intestines, kidneys, or hematopoietic cells in which Mib1-Notch signaling is required [25], [26], [27], [28], [29], [30]. When we transplanted fetal liver cells from *Snx5^+/+^* and *Snx5^-/-^* mice into lethally irradiated recipient mice, we did not observe any developmental alteration of immune cells, including T and marginal zone B lymphocytes (data not shown) [26], [28]. Moreover, in this experiment, E18.5 *Snx5^-/-^* lungs did not show a significant alteration in Notch signaling-related genes, such as *Hes1*, *Hes5*, *Hey1*, *Hey2*, and *HeyL*, as assessed by qRT-PCR (data not shown). Thus, we conclude that disruption of the *Snx5* gene in lungs does not alter Notch signaling.

In summary, this study demonstrated that deletion of the *Snx5* gene in mice can cause neonatal death, most likely due to saccular immaturity. Snx5 is essential for the differentiation and maturation of distal alveolar epithelial type I cells during lung development. Although the underlying mechanisms of Snx5 in lung development are unknown, it is plausible that Snx5 mediates lung maturity. Future studies of Snx5 with new binding molecules will deepen our understanding of the critical mechanisms involved in mouse lung development and provide new insight into the functions of SNXs in the cellular regulation of endocytic trafficking. Moreover, elucidation of the function of Snx5 in lung development may provide targets for novel therapeutic strategies for the prevention and treatment of respiratory distress syndrome.

## Supporting Information

Figure S1 **Characterization of** ***Snx5*** **-trapped mice.** (A) Southern blot analysis of gene-trapped mouse tails. The wild-type (WT) allele (4.2 kb) and the mutant (MT) allele (5.8 kb) were generated by *Eco*R1 restriction enzyme digestion. (B) PCR-based genotype analysis of the progenies of *Snx5* heterozygous crosses. The WT band was detected using W-L (exon7) and W-R (exon7) primers (Figure 1A). The MT band was detected using W-L (exon7) and M-R (β-geo cassette) primers. (C) RT-PCR and (D) western blot of mouse lung tissue. Mouse *Snx5* mRNA and protein were absent in tissue derived from *Snx5^-/-^* mice. (E) A representative growth retardation phenotype in *Snx5^-/-^* mice at P30. (F) Graph shows a reduced body weight of *Snx5^-/-^* mice at P21.
(TIF)

Figure S2 **Normal structure of the respiratory organs and heart.** (A-J) Hematoxylin and Eosin (H&E) staining of respiratory organs at E18.5. (A and B) Palates, (C–F) morphology of sagittal and transverse sections of the trachea and esophagus, (G and H) diaphragm and (I and J) intercostals muscles showing similar structures in *Snx5^-/-^* mice and controls. (A and B) Scale bars: 1 mm, (C and D) scale bars: 400 µm, (E and F) scale bars: 200 µm, (G–J) scale bars: 100 µm. (K and L) Light micrograph of the separated trachea and esophagus in E18.5 *Snx5^-/-^* mice. Scale bars: 1 mm. (M and N) Alcian blue staining of tracheal cartilage at E18.5. Scale bars: 0.5 mm. (O and P) Normal morphological structure of *Snx5^-/-^* heart compared to controls. (O and P) Scale bars: 1 mm.
(TIF)

Figure S3 **Proliferation was not altered in** ***Snx5^-/-^*** **mice.** (A) Ki67 and Hoechst double staining of *Snx5^+/+^* and *Snx5^-/-^* lungs at E18.5. Scale bars: 50 µm. (B) Graph of Ki67-positive cell counts showed similar proliferation rates in *Snx5^-/-^* and *Snx5^+/+^* lungs. Ki67-positive cells were counted in 3 random 400× microscope fields. Means ± SDs were determined using 4 *Snx5^+/+^* embryos and 7 *Snx5^-/-^* embryos in each group.
(TIF)

Figure S4 **Mature SP-B and lung Sat PC content were not decreased in** ***Snx5^-/-^*** **mice.** (A) Relative density of SP-B western blotting was quantified using densitometry (n  =  5). (B) Western blotting analysis of mature SP-B in 15 µl lung homogenate supernatant (n  =  5). Supernatant was recovered from lung homogenate after centrifugation at 1500xg for 15 min. (C) Lung Sat PC content and Sat PC to body weight levels in *Snx5^+/+^*, *Snx5^+/-^*, and *Snx5^-/-^* mice (n  =  6, *P < 0.05).
(TIF)

Figure S5 **Ultrastructure of alveolar epithelial type I cells in** ***Snx5^-/-^*** **lungs. (**A-D) Transmission electron microscopy (TEM) was used to determine ultrastructural morphology of the alveolar epithelial type I cells in *Snx5^-/-^* and *Snx5^+/+^* lungs at E18.5. (A and B) Vesicle (Vs; black arrow) and mitochondria (Mt; white arrowhead) were observed in *Snx5^+/+^* (A) and *Snx5^-/-^* (B) lungs. Scale bars: 2 µm. (C and D) Extended cytoplasm was also observed in *Snx5^+/+^* (C) and *Snx5^-/-^* (D) mice. Scale bars: 400 nm.
(TIF)

Figure S6 **Identification of primary lung epithelial cell (PLE) characteristics. (**A) Western blot analysis of lysates (10 µg) from MEFs and isolated PLEs. Isolated PLEs exhibited E-cadherin but not vimentin expression. (B) Mean relative log_10_ mRNA expression for vimentin, E-cadherin, SP-C, and T1α from MEFs and PLEs using qRT-PCR. Graphs revealed dramatic increases in E-cadherin and SP-C expression in isolated PLEs. Also, T1α was expressed at low levels in PLEs compared to the negative control, MEFs. (C) Bright-field and fluorescence images of PLEs. Bright-field images showed a type II epithelial cell-like morphology. Fluorescence images revealed partial T1α and strong proSP-C expression. Scale bars: 100 µm.
(TIF)

Figure S7 **EGFR expression in E18.5** ***Snx5^-/-^*** **lungs was not altered.** (A) Western blotting of EGFR protein in whole-lung lysates from E18.5 *Snx5^+/+^* and *Snx5^-/-^* mice (n  =  10 each). (B) Densitometric analysis showed that the ratio of EGFR to β-tubulin was similar between E18.5 *Snx5^-/-^* and *Snx5^+/+^* lungs.
(TIF)

Table S1 Biological processes enriched with up or down-regulated DEGs of E18.5 *Snx5^-/-^* lungs. Genome-wide analysis of E18.5 *Snx5^-/-^* lungs showed the biological processes enriched with up or down-regulated DEGs.
(TIF)

Table S2 Used primers for qRT-PCR in lungs. The qRT-PCR primers for analysis of changed mRNA expression levels in *Snx5^-/-^* compared to *Snx5^+/+^* lung.
(TIF)

## References

[1] Morrisey EE, Hogan BL (2010) Preparing for the first breath: genetic and cellular mechanisms in lung development. Dev Cell 18: 8–23. 20152174 10.1016/j.devcel.2009.12.010 PMC3736813

[2] Hogan BL (1999) Morphogenesis. Cell 96: 225–233. 9988217 10.1016/s0092-8674(00)80562-0

[3] Costa RH, Kalinichenko VV, Lim L (2001) Transcription factors in mouse lung development and function. Am J Physiol Lung Cell Mol Physiol 280: L823–838. 11290504 10.1152/ajplung.2001.280.5.L823

[4] Kumar VH, Lakshminrusimha S, El Abiad MT, Chess PR, Ryan RM (2005) Growth factors in lung development. Adv Clin Chem 40: 261–316. 16355925 10.1016/s0065-2423(05)40007-4

[5] Rawlins EL (2011) The building blocks of mammalian lung development. Dev Dyn 240: 463–476. 21337459 10.1002/dvdy.22482

[6] Warburton D, Wuenschell C, Flores-Delgado G, Anderson K (1998) Commitment and differentiation of lung cell lineages. Biochem Cell Biol 76: 971–995. 10392710

[7] Williams MC (2003) Alveolar type I cells: molecular phenotype and development. Annu Rev Physiol 65: 669–695. 12428023 10.1146/annurev.physiol.65.092101.142446

[8] Cole FS, Hamvas A, Nogee LM (2001) Genetic disorders of neonatal respiratory function. Pediatr Res 50: 157–162. 11477198 10.1203/00006450-200108000-00001

[9] Glasser SW, Nogee LM (2006) Genetically engineered mice in understanding the basis of neonatal lung disease. Semin Perinatol 30: 341–349. 17142160 10.1053/j.semperi.2006.09.001

[10] Lyra PP, Diniz EM (2007) The importance of surfactant on the development of neonatal pulmonary diseases. Clinics (Sao Paulo) 62: 181–190. 17505704 10.1590/s1807-59322007000200014

[11] Clark JC, Wert SE, Bachurski CJ, Stahlman MT, Stripp BR, et al (1995) Targeted disruption of the surfactant protein B gene disrupts surfactant homeostasis, causing respiratory failure in newborn mice. Proc Natl Acad Sci U S A 92: 7794–7798. 7644495 10.1073/pnas.92.17.7794 PMC41232

[12] deMello DE, Heyman S, Phelps DS, Hamvas A, Nogee L, et al (1994) Ultrastructure of lung in surfactant protein B deficiency. Am J Respir Cell Mol Biol 11: 230–239. 8049084 10.1165/ajrcmb.11.2.8049084

[13] Glasser SW, Burhans MS, Korfhagen TR, Na CL, Sly PD, et al (2001) Altered stability of pulmonary surfactant in SP-C-deficient mice. Proc Natl Acad Sci U S A 98: 6366–6371. 11344267 10.1073/pnas.101500298 PMC33474

[14] Ban N, Matsumura Y, Sakai H, Takanezawa Y, Sasaki M, et al (2007) ABCA3 as a lipid transporter in pulmonary surfactant biogenesis. J Biol Chem 282: 9628–9634. 17267394 10.1074/jbc.M611767200

[15] Pei L, Leblanc M, Barish G, Atkins A, Nofsinger R, et al (2011) Thyroid hormone receptor repression is linked to type I pneumocyte-associated respiratory distress syndrome. Nat Med 17: 1466–1472. 22001906 10.1038/nm.2450 PMC3210920

[16] Galambos C, Ng YS, Ali A, Noguchi A, Lovejoy S, et al (2002) Defective pulmonary development in the absence of heparin-binding vascular endothelial growth factor isoforms. Am J Respir Cell Mol Biol 27: 194–203. 12151311 10.1165/ajrcmb.27.2.4703

[17] Wan H, Xu Y, Ikegami M, Stahlman MT, Kaestner KH, et al (2004) Foxa2 is required for transition to air breathing at birth. Proc Natl Acad Sci U S A 101: 14449–14454. 15452354 10.1073/pnas.0404424101 PMC521955

[18] Liu H, Liu ZQ, Chen CX, Magill S, Jiang Y, et al (2006) Inhibitory regulation of EGF receptor degradation by sorting nexin 5. Biochem Biophys Res Commun 342: 537–546. 16487940 10.1016/j.bbrc.2006.01.179

[19] Merino-Trigo A, Kerr MC, Houghton F, Lindberg A, Mitchell C, et al (2004) Sorting nexin 5 is localized to a subdomain of the early endosomes and is recruited to the plasma membrane following EGF stimulation. J Cell Sci 117: 6413–6424. 15561769 10.1242/jcs.01561

[20] Wassmer T, Attar N, Bujny MV, Oakley J, Traer CJ, et al (2007) A loss-of-function screen reveals SNX5 and SNX6 as potential components of the mammalian retromer. J Cell Sci 120: 45–54. 17148574 10.1242/jcs.03302

[21] Yoo KW, Kim EH, Jung SH, Rhee M, Koo BK, et al (2006) Snx5, as a Mind bomb-binding protein, is expressed in hematopoietic and endothelial precursor cells in zebrafish. FEBS Lett 580: 4409–4416. 16857196 10.1016/j.febslet.2006.07.009

[22] Carlton J, Bujny M, Peter BJ, Oorschot VM, Rutherford A, et al (2004) Sorting nexin-1 mediates tubular endosome-to-TGN transport through coincidence sensing of high- curvature membranes and 3-phosphoinositides. Curr Biol 14: 1791–1800. 15498486 10.1016/j.cub.2004.09.077

[23] Cavet ME, Pang J, Yin G, Berk BC (2008) An epidermal growth factor (EGF) -dependent interaction between GIT1 and sorting nexin 6 promotes degradation of the EGF receptor. FASEB J 22: 3607–3616. 18523162 10.1096/fj.07-094086 PMC2537429

[24] McGough IJ, Cullen PJ (2011) Recent advances in retromer biology. Traffic 12: 963–971. 21463457 10.1111/j.1600-0854.2011.01201.x

[25] Jeong HW, Jeon US, Koo BK, Kim WY, Im SK, et al (2009) Inactivation of Notch signaling in the renal collecting duct causes nephrogenic diabetes insipidus in mice. J Clin Invest 119: 3290–3300. 19855135 10.1172/JCI38416 PMC2769200

[26] Kim YW, Koo BK, Jeong HW, Yoon MJ, Song R, et al (2008) Defective Notch activation in microenvironment leads to myeloproliferative disease. Blood 112: 4628–4638. 18818392 10.1182/blood-2008-03-148999

[27] Koo BK, Lim HS, Song R, Yoon MJ, Yoon KJ, et al (2005) Mind bomb 1 is essential for generating functional Notch ligands to activate Notch. Development 132: 3459–3470. 16000382 10.1242/dev.01922

[28] Song R, Kim YW, Koo BK, Jeong HW, Yoon MJ, et al (2008) Mind bomb 1 in the lymphopoietic niches is essential for T and marginal zone B cell development. J Exp Med 205: 2525–2536. 18824586 10.1084/jem.20081344 PMC2571928

[29] Yoon KJ, Koo BK, Im SK, Jeong HW, Ghim J, et al (2008) Mind bomb 1-expressing intermediate progenitors generate notch signaling to maintain radial glial cells. Neuron 58: 519–531. 18498734 10.1016/j.neuron.2008.03.018

[30] Yoon MJ, Koo BK, Song R, Jeong HW, Shin J, et al (2008) Mind bomb-1 is essential for intraembryonic hematopoiesis in the aortic endothelium and the subaortic patches. Mol Cell Biol 28: 4794–4804. 18505817 10.1128/MCB.00436-08 PMC2493361

[31] Weinmaster G, Fischer JA (2011) Notch ligand ubiquitylation: what is it good for? Dev Cell 21: 134–144. 21763614 10.1016/j.devcel.2011.06.006 PMC3156059

[32] Warshamana GS, Corti M, Brody AR (2001) TNF-alpha, PDGF, and TGF-beta(1) expression by primary mouse bronchiolar-alveolar epithelial and mesenchymal cells: tnf-alpha induces TGF-beta(1). Exp Mol Pathol 71: 13–33. 11502094 10.1006/exmp.2001.2376

[33] Matsui R, Brody JS, Yu Q (1999) FGF-2 induces surfactant protein gene expression in foetal rat lung epithelial cells through a MAPK-independent pathway. Cell Signal 11: 221–228. 10353697 10.1016/s0898-6568(98)00070-9

[34] Mason RJ, Nellenbogen J, Clements JA (1976) Isolation of disaturated phosphatidylcholine with osmium tetroxide. J Lipid Res 17: 281–284. 932560

[35] Martis PC, Whitsett JA, Xu Y, Perl AK, Wan H, et al (2006) C/EBPalpha is required for lung maturation at birth. Development 133: 1155–1164. 16467360 10.1242/dev.02273

[36] Bartlett GR (1959) Phosphorus assay in column chromatography. J Biol Chem 234: 466–468. 13641241

[37] Geng Y, Dong Y, Yu M, Zhang L, Yan X, et al (2011) Follistatin-like 1 (Fstl1) is a bone morphogenetic protein (BMP) 4 signaling antagonist in controlling mouse lung development. Proc Natl Acad Sci U S A 108: 7058–7063. 21482757 10.1073/pnas.1007293108 PMC3084141

[38] Matzke A, Sargsyan V, Holtmann B, Aramuni G, Asan E, et al (2007) Haploinsufficiency of c-Met in cd44-/- mice identifies a collaboration of CD44 and c-Met in vivo. Mol Cell Biol 27: 8797–8806. 17923692 10.1128/MCB.01355-07 PMC2169422

[39] Jin N, Cho SN, Raso MG, Wistuba I, Smith Y, et al (2009) Mig-6 is required for appropriate lung development and to ensure normal adult lung homeostasis. Development 136: 3347–3356. 19710174 10.1242/dev.032979 PMC2739148

[40] Compernolle V, Brusselmans K, Acker T, Hoet P, Tjwa M, et al (2002) Loss of HIF-2alpha and inhibition of VEGF impair fetal lung maturation, whereas treatment with VEGF prevents fatal respiratory distress in premature mice. Nat Med 8: 702–710. 12053176 10.1038/nm721

[41] Bolstad BM, Irizarry RA, Astrand M, Speed TP (2003) A comparison of normalization methods for high density oligonucleotide array data based on variance and bias. Bioinformatics 19: 185–193. 12538238 10.1093/bioinformatics/19.2.185

[42] Lee JS, Kim Y, Kim IS, Kim B, Choi HJ, et al (2010) Negative regulation of hypoxic responses via induced Reptin methylation. Mol Cell 39: 71–85. 20603076 10.1016/j.molcel.2010.06.008 PMC4651011

[43] Hwang D, Smith JJ, Leslie DM, Weston AD, Rust AG, et al (2005) A data integration methodology for systems biology: experimental verification. Proc Natl Acad Sci U S A 102: 17302–17307. 16301536 10.1073/pnas.0508649102 PMC1297683

[44] Wert SE, Whitsett JA, Nogee LM (2009) Genetic disorders of surfactant dysfunction. Pediatr Dev Pathol 12: 253–274. 19220077 10.2350/09-01-0586.1 PMC2987676

[45] Andreeva AV, Kutuzov MA, Voyno-Yasenetskaya TA (2007) Regulation of surfactant secretion in alveolar type II cells. Am J Physiol Lung Cell Mol Physiol 293: L259–271. 17496061 10.1152/ajplung.00112.2007

[46] McElroy MC, Kasper M (2004) The use of alveolar epithelial type I cell-selective markers to investigate lung injury and repair. Eur Respir J 24: 664–673. 15459148 10.1183/09031936.04.00096003

[47] Hara S, Kiyokawa E, Iemura S, Natsume T, Wassmer T, et al (2008) The DHR1 domain of DOCK180 binds to SNX5 and regulates cation-independent mannose 6-phosphate receptor transport. Mol Biol Cell 19: 3823–3835. 18596235 10.1091/mbc.E08-03-0314 PMC2526700

[48] Seaman MN (2004) Cargo-selective endosomal sorting for retrieval to the Golgi requires retromer. J Cell Biol 165: 111–122. 15078902 10.1083/jcb.200312034 PMC2172078

[49] Panaro NJ, Yuen PK, Sakazume T, Fortina P, Kricka LJ, et al (2000) Evaluation of DNA fragment sizing and quantification by the agilent 2100 bioanalyzer. Clin Chem 46: 1851–1853. 11067828

[50] Kalin TV, Wang IC, Meliton L, Zhang Y, Wert SE, et al (2008) Forkhead Box m1 transcription factor is required for perinatal lung function. Proc Natl Acad Sci U S A 105: 19330–19335. 19033457 10.1073/pnas.0806748105 PMC2587228

[51] Klinger S, Turgeon B, Levesque K, Wood GA, Aagaard-Tillery KM, et al (2009) Loss of Erk3 function in mice leads to intrauterine growth restriction, pulmonary immaturity, and neonatal lethality. Proc Natl Acad Sci U S A 106: 16710–16715. 19805361 10.1073/pnas.0900919106 PMC2757836

[52] O'Brien KB, Alberich-Jorda M, Yadav N, Kocher O, Diruscio A, et al (2010) CARM1 is required for proper control of proliferation and differentiation of pulmonary epithelial cells. Development 137: 2147–2156. 20530543 10.1242/dev.037150 PMC2882134

[53] Schacht V, Ramirez MI, Hong YK, Hirakawa S, Feng D, et al (2003) T1alpha/podoplanin deficiency disrupts normal lymphatic vasculature formation and causes lymphedema. EMBO J 22: 3546–3556. 12853470 10.1093/emboj/cdg342 PMC165612

[54] Miettinen PJ, Berger JE, Meneses J, Phung Y, Pedersen RA, et al (1995) Epithelial immaturity and multiorgan failure in mice lacking epidermal growth factor receptor. Nature 376: 337–341. 7630400 10.1038/376337a0

[55] Griffin CT, Trejo J, Magnuson T (2005) Genetic evidence for a mammalian retromer complex containing sorting nexins 1 and 2. Proc Natl Acad Sci U S A 102: 15173–15177. 16214895 10.1073/pnas.0409558102 PMC1257690

[56] Verges M (2007) Retromer and sorting nexins in development. Front Biosci 12: 3825–3851. 17485342 10.2741/2355

