# Supplementary figures and images for: Disruption of Sorting Nexin 5 Causes Respiratory Failure Associated with Undifferentiated Alveolar Epithelial Type I Cells in Mice
Source: PLoS One. 2013 Mar 19;8(3):e58511. doi: 10.1371/journal.pone.0058511 (PMC3602295; doi:10.1371/journal.pone.0058511)

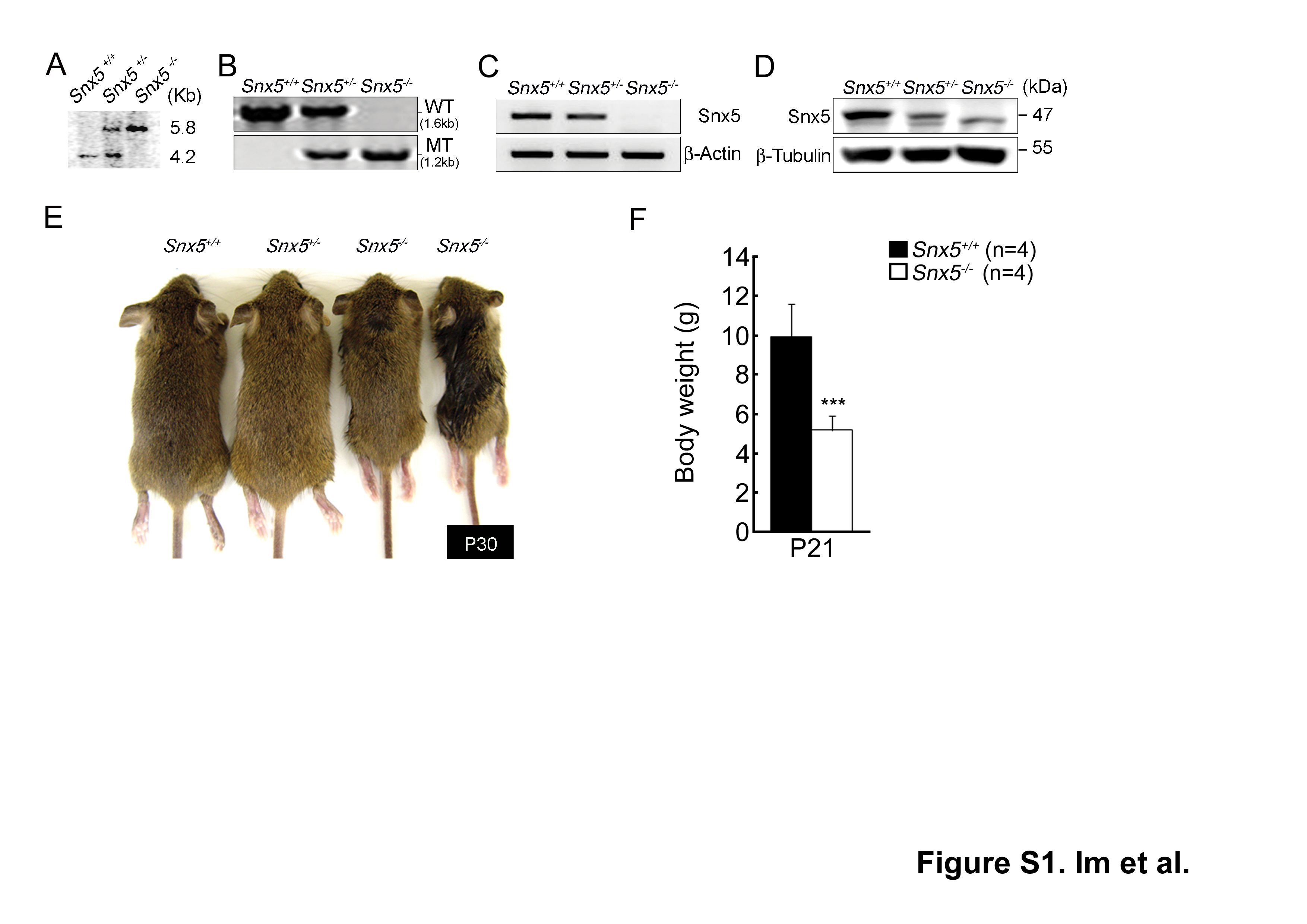

Supplement: Figure S1 — Characterization of Snx5 -trapped mice. (A) Southern blot analysis of gene-trapped mouse tails. The wild-type (WT) allele (4.2 kb) and the mutant (MT) allele (5.8 kb) were generated by EcoR1 restriction enzyme digestion. (B) PCR-based genotype analysis of the progenies of Snx5 heterozygous crosses. The WT band was detected using W-L (exon7) and W-R (exon7) primers (Figure 1A). The MT band was detected using W-L (exon7) and M-R (β-geo cassette) primers. (C) RT-PCR and (D) western blot of mouse lung tissue. Mouse Snx5 mRNA and protein were absent in tissue derived from Snx5-/- mice. (E) A representative growth retardation phenotype in Snx5-/- mice at P30. (F) Graph shows a reduced body weight of Snx5-/- mice at P21. (TIF) [file pone.0058511.s001.tif]

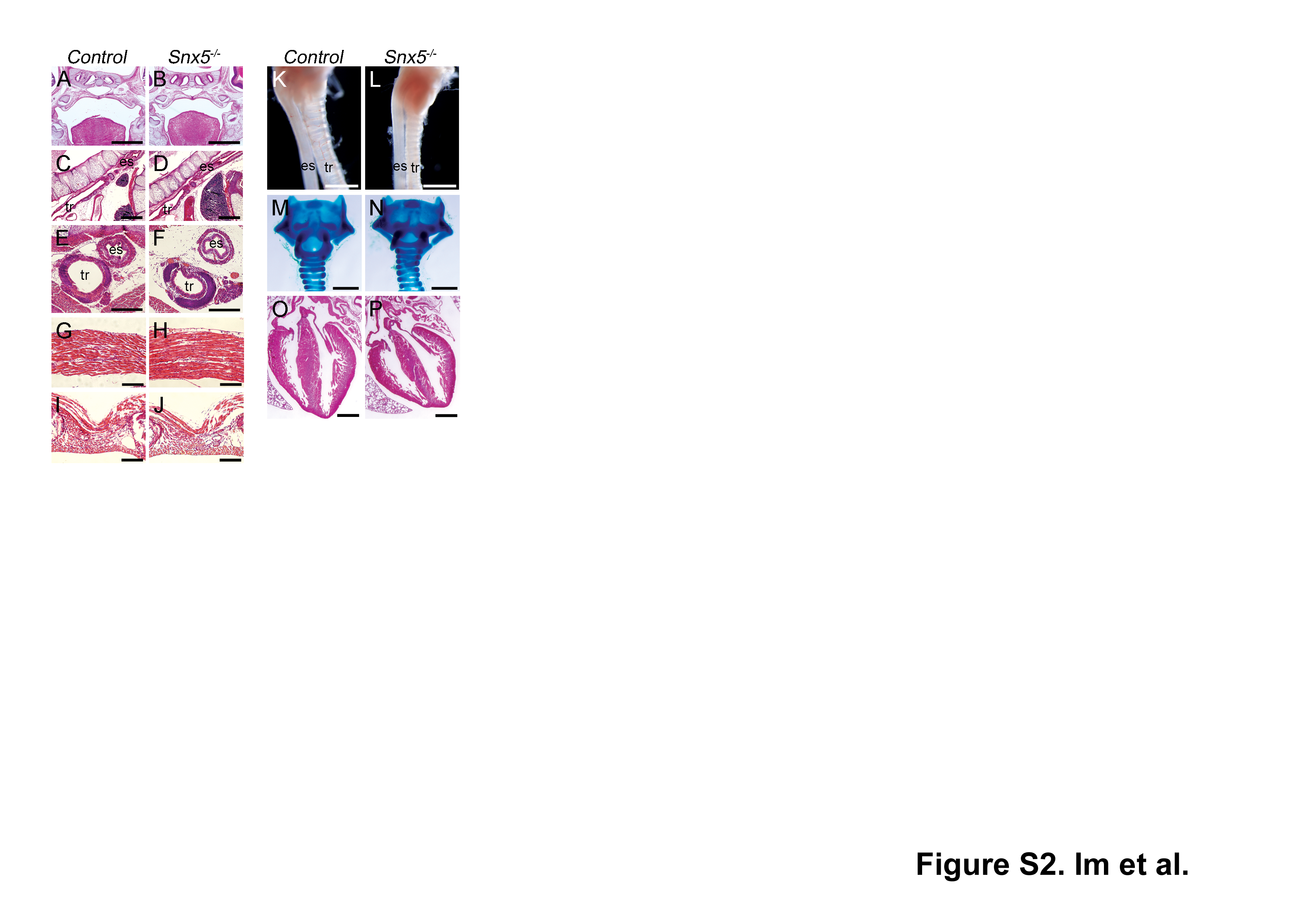

Supplement: Figure S2 — Normal structure of the respiratory organs and heart. (A-J) Hematoxylin and Eosin (H&E) staining of respiratory organs at E18.5. (A and B) Palates, (C–F) morphology of sagittal and transverse sections of the trachea and esophagus, (G and H) diaphragm and (I and J) intercostals muscles showing similar structures in Snx5-/- mice and controls. (A and B) Scale bars: 1 mm, (C and D) scale bars: 400 µm, (E and F) scale bars: 200 µm, (G–J) scale bars: 100 µm. (K and L) Light micrograph of the separated trachea and esophagus in E18.5 Snx5-/- mice. Scale bars: 1 mm. (M and N) Alcian blue staining of tracheal cartilage at E18.5. Scale bars: 0.5 mm. (O and P) Normal morphological structure of Snx5-/- heart compared to controls. (O and P) Scale bars: 1 mm. (TIF) [file pone.0058511.s002.tif]

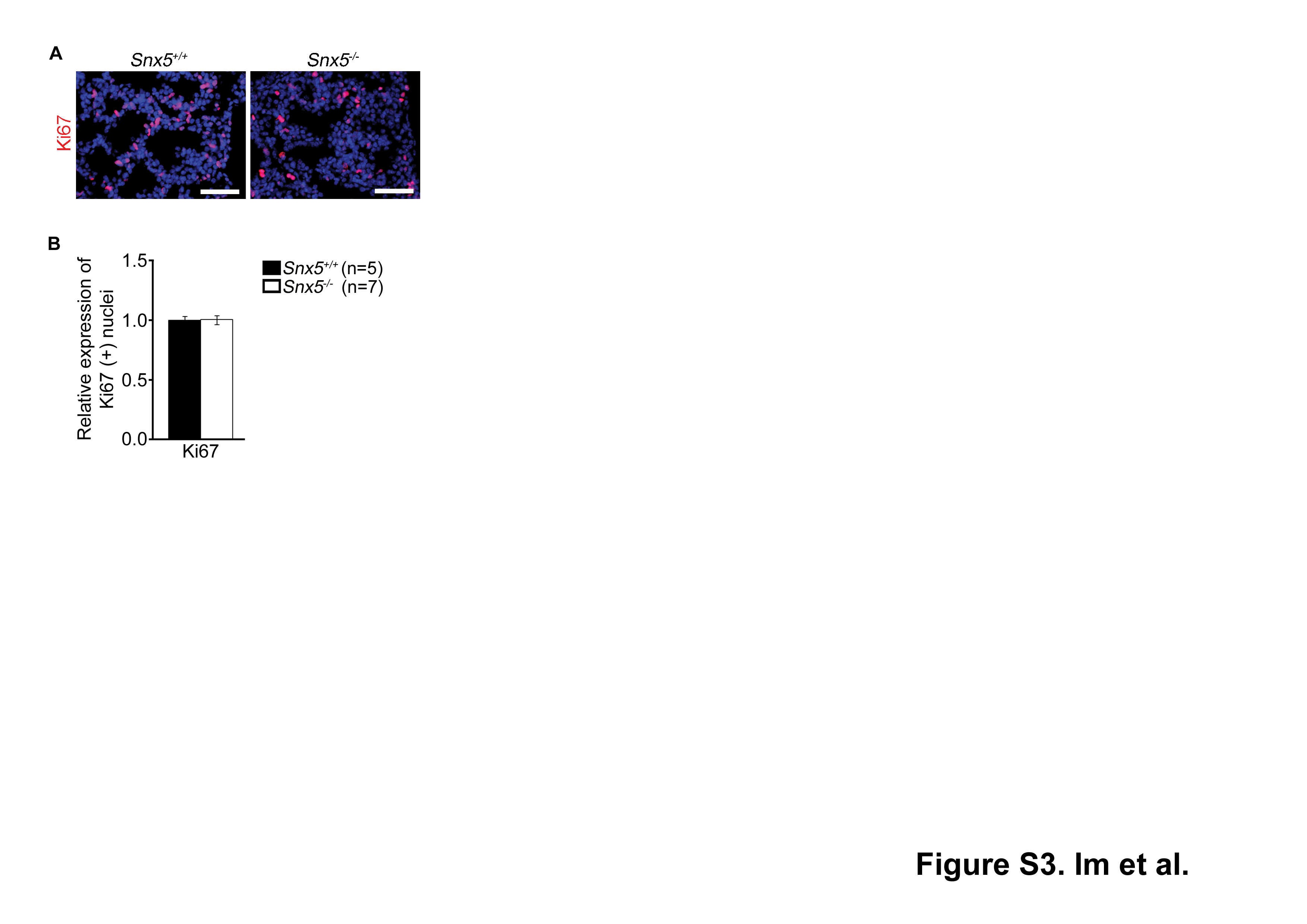

Supplement: Figure S3 — Proliferation was not altered in Snx5-/- mice. (A) Ki67 and Hoechst double staining of Snx5+/+ and Snx5-/- lungs at E18.5. Scale bars: 50 µm. (B) Graph of Ki67-positive cell counts showed similar proliferation rates in Snx5-/- and Snx5+/+ lungs. Ki67-positive cells were counted in 3 random 400× microscope fields. Means ± SDs were determined using 4 Snx5+/+ embryos and 7 Snx5-/- embryos in each group. (TIF) [file pone.0058511.s003.tif]

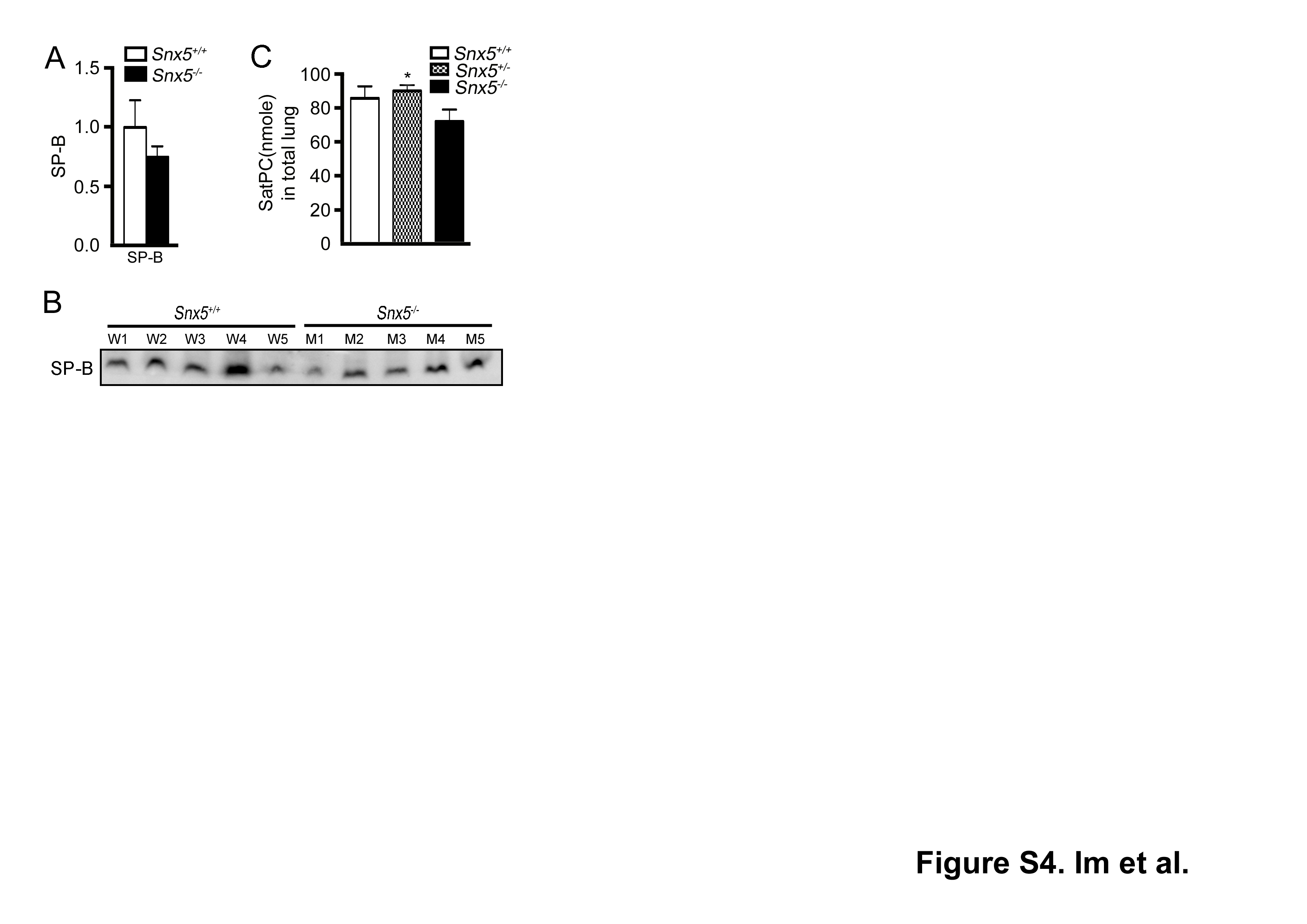

Supplement: Figure S4 — Mature SP-B and lung Sat PC content were not decreased in Snx5-/- mice. (A) Relative density of SP-B western blotting was quantified using densitometry (n = 5). (B) Western blotting analysis of mature SP-B in 15 µl lung homogenate supernatant (n = 5). Supernatant was recovered from lung homogenate after centrifugation at 1500xg for 15 min. (C) Lung Sat PC content and Sat PC to body weight levels in Snx5+/+, Snx5+/-, and Snx5-/- mice (n = 6, *P < 0.05). (TIF) [file pone.0058511.s004.tif]

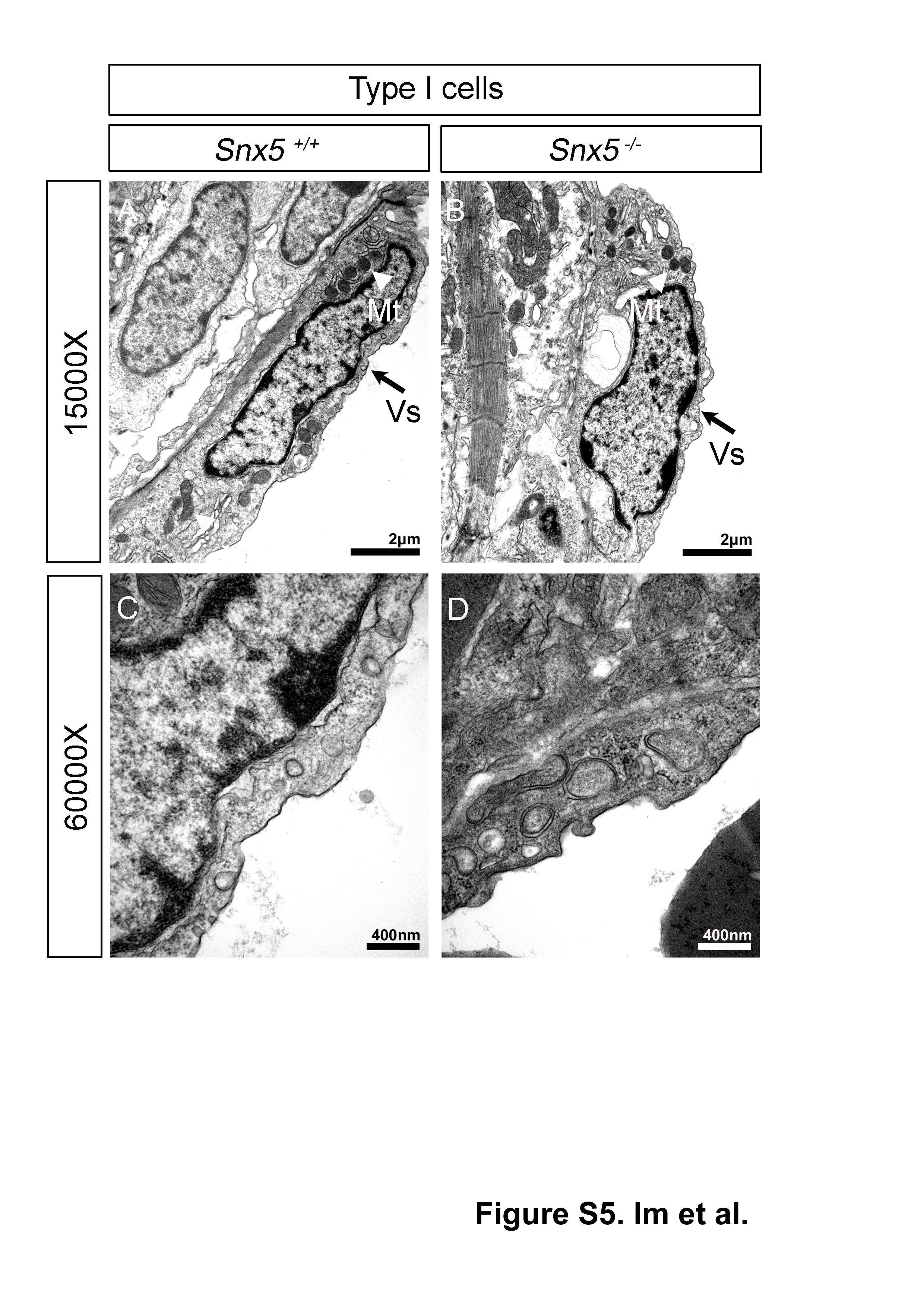

Supplement: Figure S5 — Ultrastructure of alveolar epithelial type I cells in Snx5-/- lungs. (A-D) Transmission electron microscopy (TEM) was used to determine ultrastructural morphology of the alveolar epithelial type I cells in Snx5-/- and Snx5+/+ lungs at E18.5. (A and B) Vesicle (Vs; black arrow) and mitochondria (Mt; white arrowhead) were observed in Snx5+/+ (A) and Snx5-/- (B) lungs. Scale bars: 2 µm. (C and D) Extended cytoplasm was also observed in Snx5+/+ (C) and Snx5-/- (D) mice. Scale bars: 400 nm. (TIF) [file pone.0058511.s005.tif]

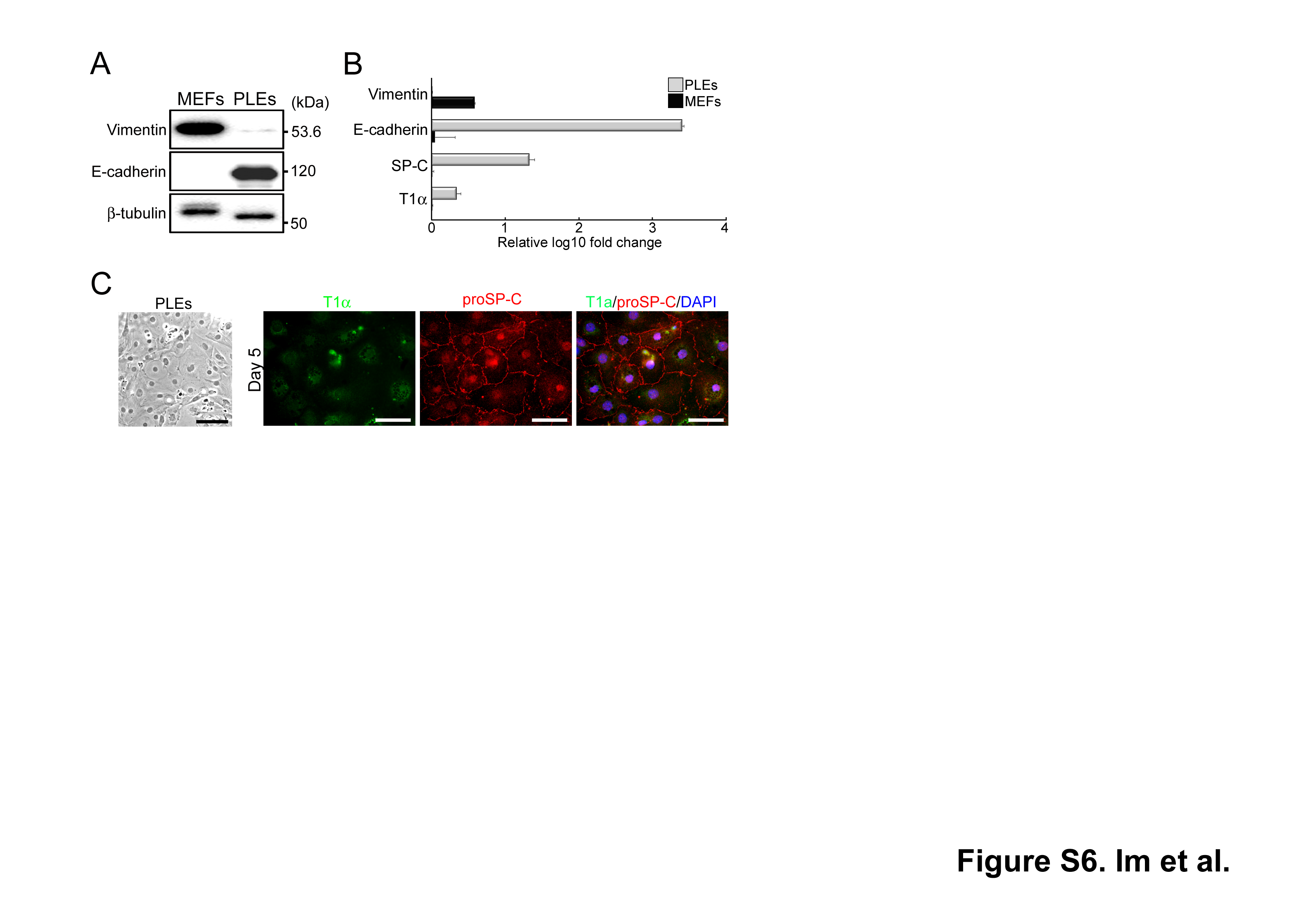

Supplement: Figure S6 — Identification of primary lung epithelial cell (PLE) characteristics. (A) Western blot analysis of lysates (10 µg) from MEFs and isolated PLEs. Isolated PLEs exhibited E-cadherin but not vimentin expression. (B) Mean relative log10 mRNA expression for vimentin, E-cadherin, SP-C, and T1α from MEFs and PLEs using qRT-PCR. Graphs revealed dramatic increases in E-cadherin and SP-C expression in isolated PLEs. Also, T1α was expressed at low levels in PLEs compared to the negative control, MEFs. (C) Bright-field and fluorescence images of PLEs. Bright-field images showed a type II epithelial cell-like morphology. Fluorescence images revealed partial T1α and strong proSP-C expression. Scale bars: 100 µm. (TIF) [file pone.0058511.s006.tif]

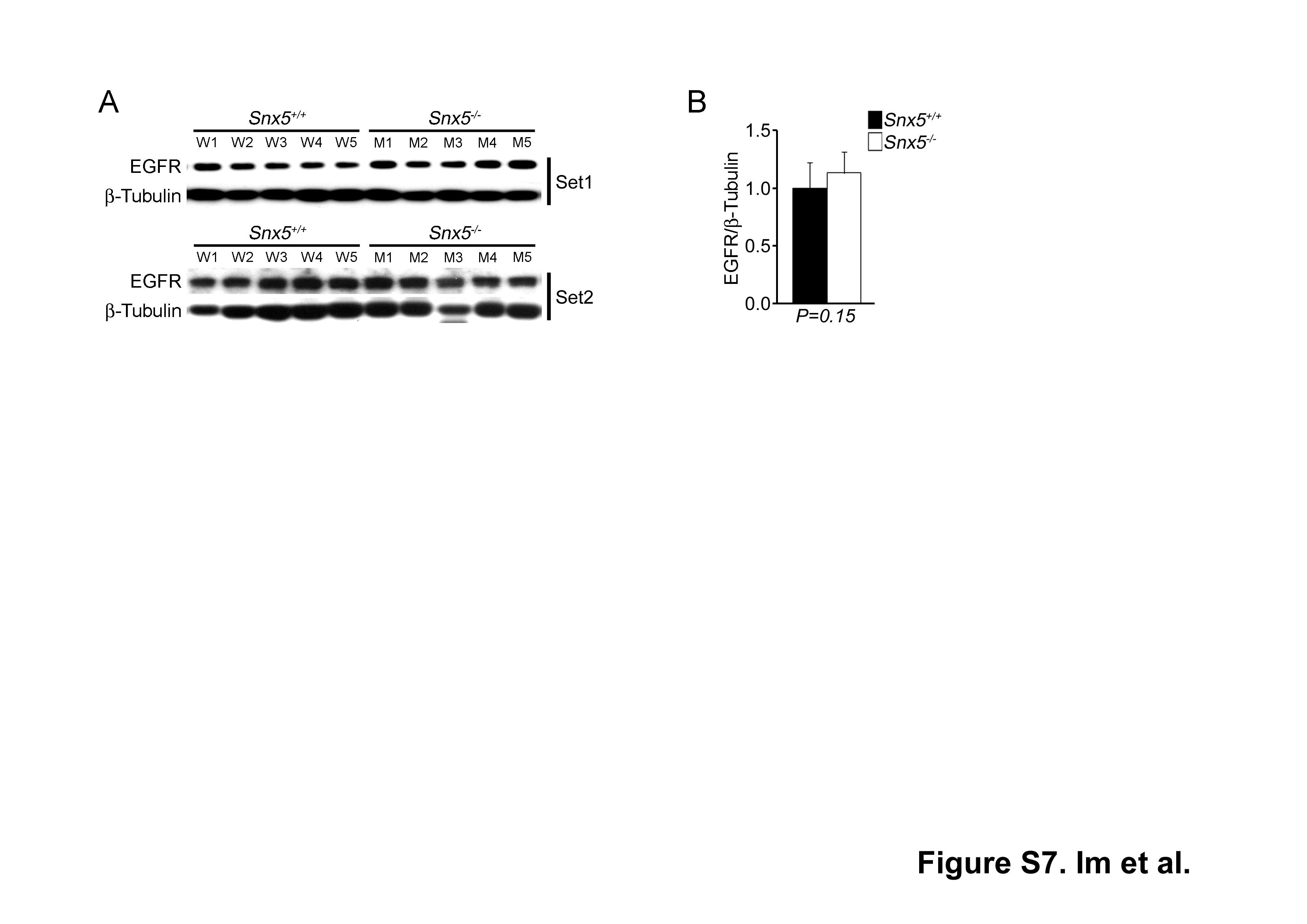

Supplement: Figure S7 — EGFR expression in E18.5 Snx5-/- lungs was not altered. (A) Western blotting of EGFR protein in whole-lung lysates from E18.5 Snx5+/+ and Snx5-/- mice (n = 10 each). (B) Densitometric analysis showed that the ratio of EGFR to β-tubulin was similar between E18.5 Snx5-/- and Snx5+/+ lungs. (TIF) [file pone.0058511.s007.tif]

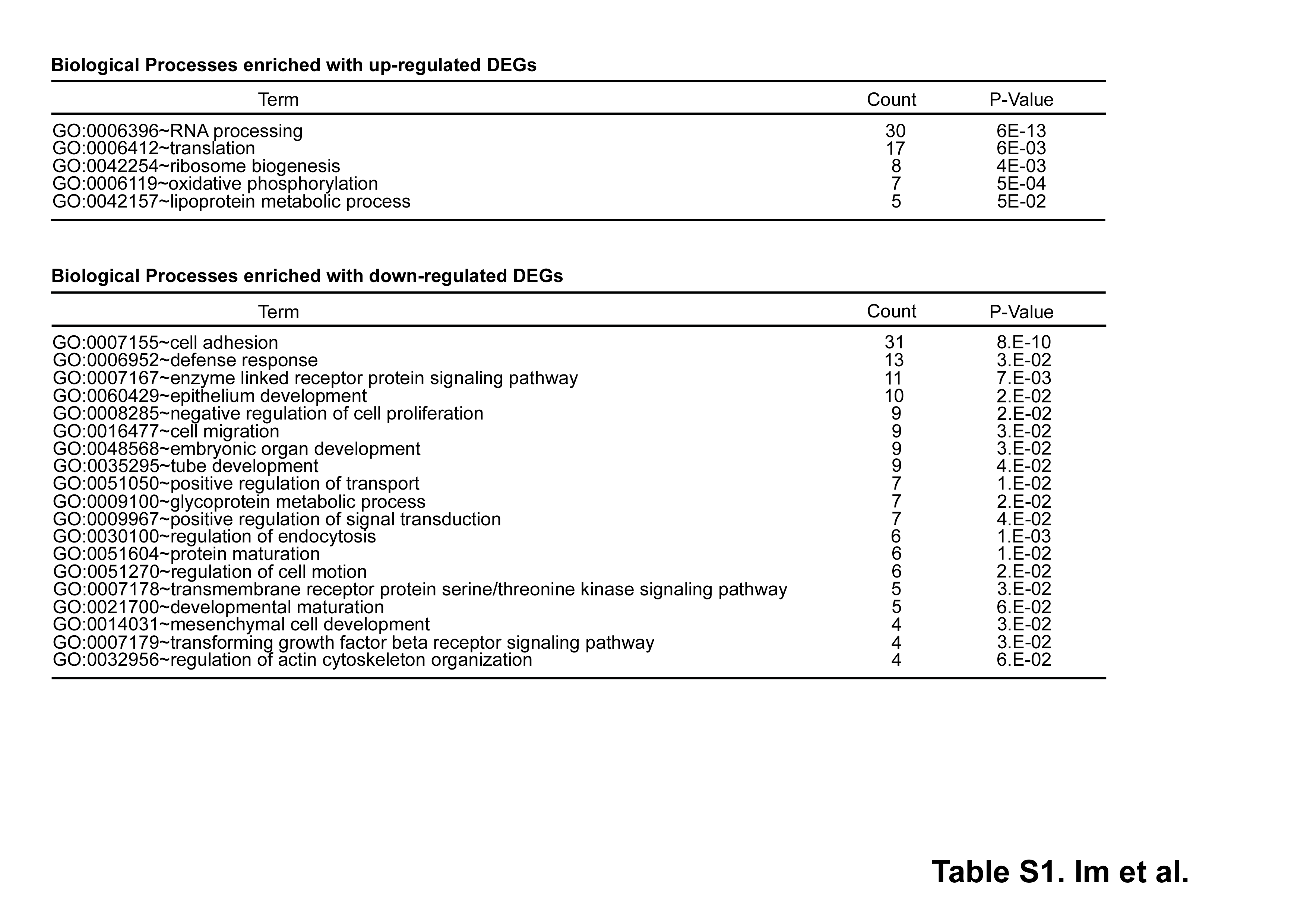

Supplement: Table S1 — Biological processes enriched with up or down-regulated DEGs of E18.5 Snx5-/- lungs. Genome-wide analysis of E18.5 Snx5-/- lungs showed the biological processes enriched with up or down-regulated DEGs. (TIF) [file pone.0058511.s008.tif]

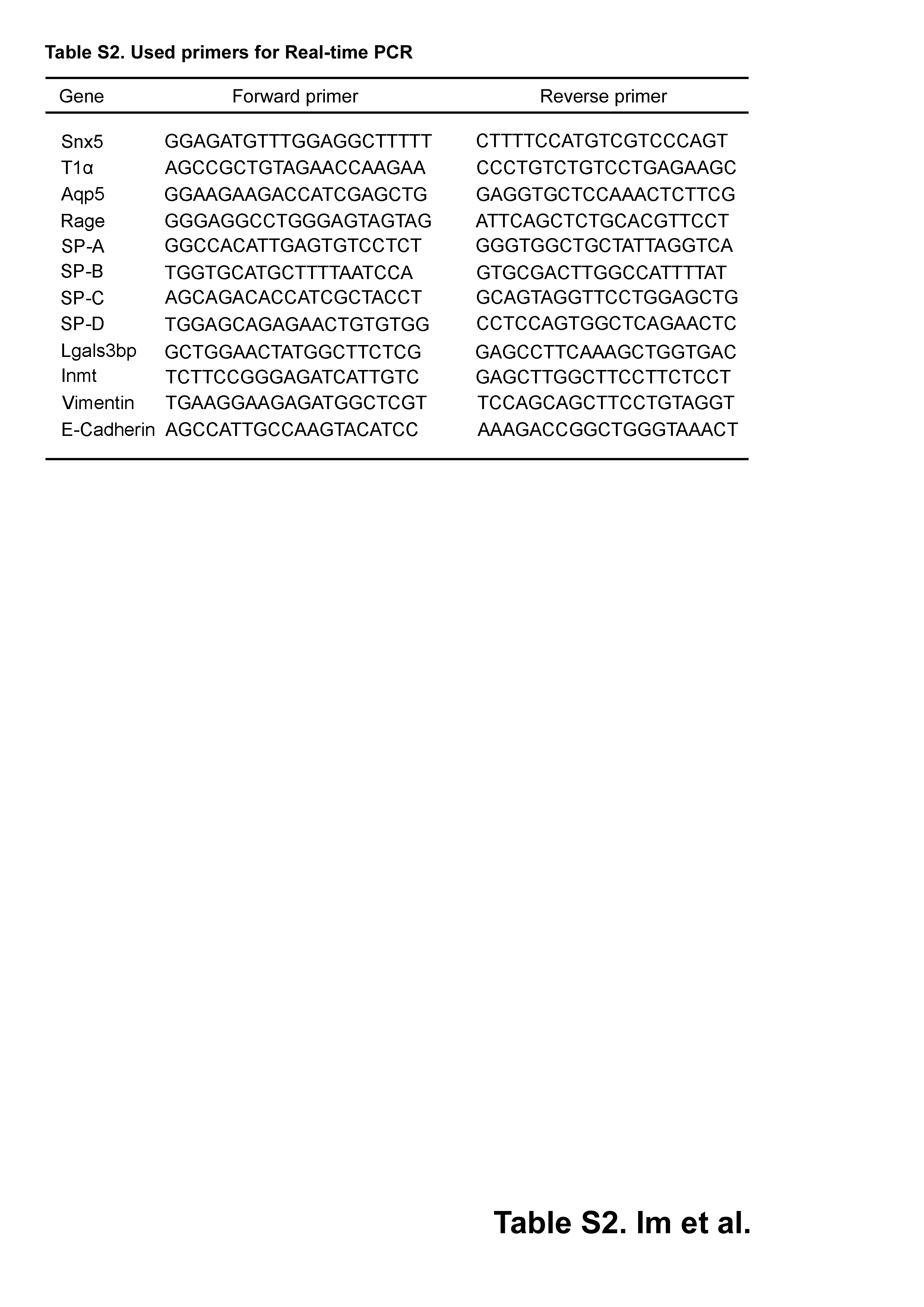

Supplement: Table S2 — Used primers for qRT-PCR in lungs. The qRT-PCR primers for analysis of changed mRNA expression levels in Snx5-/- compared to Snx5+/+ lung. (TIF) [file pone.0058511.s009.tif]
